# The Road to Elimination of Mother-to-Child Transmission of Syphilis in Malawi: A Mixed-Methods Analysis of Health System Readiness

**DOI:** 10.1097/OLQ.0000000000002359

**Published:** 2026-05-18

**Authors:** Vester Gunsaru, Rebecca Husband, Zacharia Kafuwa, Annielisa Majamanda, Christina Mchoma, Monica Malata, Amelia C. Crampin, Gladys Membe-Gadama, Luis Gadama, David Lissauer, Elizabeth Chodzaza, Catherine Bamuya, Effie Chipeta, Peter MacPherson, Michael Marks, Chelsea Morroni, Alinane Linda Nyondo-Mipando, Brynne Gilmore, Bridget Freyne

**Affiliations:** From the * Malawi-Liverpool Wellcome Research Programme, Blantyre, Malawi; † Centre for Experimental Pathogen Host Research, School of Medicine, University College Dublin, Dublin, Ireland; ‡ School of Global and Public Health, Kamuzu University of Health Sciences, Blantyre, Malawi; § Centre for Reproductive Health, Kamuzu University of Health Sciences, Blantyre, Malawi; ¶ Blantyre District Health Office, Blantyre, Malawi; ‖ Reproductive Health Directorate, Ministry of Health, Malawi; ** Malawi Epidemiology and Intervention Research Unit, Lilongwe, Malawi; †† School of Health and Wellbeing, University of Glasgow, Glasgow, Scotland, UK; ‡‡ Department of Obstetrics, Queen Elizabeth Central Hospital, Blantyre, Malawi; §§ Johns Hopkins Research Project, Blantyre, Malawi; ¶¶ Department of Women’s and Children’s Health, Institute of Life Course and Medical Sciences, University of Liverpool, Liverpool, UK; ‖‖ London School of Hygiene and Tropical Medicine, London, UK; *** Chair in Global Sexual and Reproductive Health, University of Edinburgh, Edinburgh, Scotland, UK; ††† School of Maternal and Neonatal Health, Kamuzu University of Health Sciences, Blantyre, Malawi; ‡‡‡ School of Midwifery and Health Systems, University College Dublin, Dublin, Ireland; §§§ Departments of Infectious Diseases and Global Health, Children’s Health Ireland, Dublin, Ireland; ¶¶¶ Department of Paediatrics, Kamuzu University of Health Sciences, Blantyre, Malawi

## Abstract

**Background:**

This study evaluated health system readiness in Malawi for elimination of mother-to-child transmission (EMTCT) of syphilis against World Health Organization (WHO) validation criteria by identifying key health system bottlenecks. It also developed a replicable toolkit to assess bottlenecks to syphilis EMTCT in other countries.

**Methods:**

A mixed-methods design integrated quantitative and qualitative data. A National Clinic Readiness Survey assessed coverage indicators for prevention of maternal and congenital syphilis at 58 health centers. Exit surveys included 9513 mothers at antenatal care (ANC) and 4724 at delivery across 7 sites, with additional data from 131 syphilis-positive mothers. Exit-survey data informed two modified Tanahashi bottleneck analyses assessing EMTCT process outcomes at ANC and delivery. Semistructured in-depth interviews were conducted with 29 facility staff.

**Results:**

The readiness survey identified inefficiencies and gaps in the procurement of tests and treatment. During frequent stock-outs, most sites required mothers to purchase treatment at personal cost. Variation existed in identifying potential congenital syphilis cases and recording surveillance data across centers. Tanahashi analyses showed limited coverage of maternal testing at ANC and variable coverage of maternal treatment at ANC and delivery. Interviews indicated that syphilis treatment is deprioritized relative to Human Immunodeficiency Virus (HIV) and other conditions requiring intramuscular benzathine penicillin G (IM BPG).

**Conclusions:**

Despite strong political commitment to integrated EMTCT of syphilis, HIV, and hepatitis B, persistent health system bottlenecks may delay WHO validation. This toolkit may support monitoring and strengthening EMTCT programs in Malawi and other settings.

Congenital syphilis (CS) is preventable, and yet global efforts to achieve elimination of mother-to-child transmission (EMTCT) of syphilis have fallen short. In 2022, there were an estimated 700,000 new CS cases worldwide.^1^ CS surveillance is complicated by the lack of an effective diagnostic test and the need for a broad surveillance definition.^2^ In Africa, an estimated 3% to 9% of pregnant women are affected, though limited testing and weak surveillance systems make accurate estimates of the burden challenging.^3^ Countries seeking EMTCT validation must attain >95% coverage of the three process targets in the World Health Organization (WHO)-United Nations Children’s Fund (UNICEF) Triple Elimination strategy and a CS incidence of ≤50 cases of CS per 100,000 live births.^1^ In Africa, it is estimated that only 50% to 80% of antenatal care (ANC) attendees are tested, with 65% to 90% of those testing positive receiving treatment.^3^ While the pathway to WHO validation for EMTCT of syphilis is clear, it is dependent on the quality of data and the evaluation capacity of individual countries.

Fifteen countries have achieved WHO validation for EMTCT of syphilis.^4^ EMTCT relies on interdependent factors across the health system, and health systems that have successfully attained EMTCT status are characterized by universal free ANC, integrated HIV-syphilis testing, sustained political commitment, reliable supply chains, and strong surveillance for both maternal and congenital syphilis.^4^ In its 2020 to 2027 National Strategic Plan for HIV and Acquired Immunodeficiency Syndrome, Malawi committed to the Triple Elimination initiative, yet progress remains uneven.^5^ Based on modeled health management information system (HMIS) data, rates of gestational syphilis have increased 6-fold over the last decade.^6^ In addition, untreated maternal syphilis was identified in 2% of early stage cases and 6.1% of late or unknown-stage cases at an urban referral hospital, suggesting gaps in the EMTCT pathway.^7^

This study developed a suite of data collection and analysis tools to systematically identify barriers to EMTCT in Malawi, while also providing a replicable framework to assess and monitor progress toward syphilis EMTCT in other settings.

## METHODS

We used a mixed-methods design and developed a suite of data collection and analysis tools informed by a desk review of policy documents. The research protocol was approved by the University of Malawi, College of Medicine Research and Ethics Committee and the University of Liverpool Research Ethics Committee in January 2021. Verbal consent was obtained from all women at ANC and delivery whose data were extracted from routine sources for the exit surveys. Written consent was obtained from women who tested positive for syphilis and from healthcare workers participating in qualitative interviews and the National Clinic Readiness survey.

Clinic- and individual-level data were collected from January to September 2021 at the ANC and postnatal ward of seven health facilities providing maternity care across the Northern (Karonga), Central (Lilongwe), and Southern (Blantyre) regions. Sites were in urban and peri-urban settings in the Central and Southern regions (Zingwangwa, Mpemba, South Lunzu, Bangwe HC, and Lilongwe Area 25), and in peri-urban and rural settings in the Northern region (Karonga District Hospital and Chilumba Rural Hospital). At the clinic level, data were collected via cross-sectional exit surveys. These surveys involved data abstraction from the health passports of women attending their first ANC visit (ANC1) and postpartum women seen just after delivery at the clinic. A separate patient-reported exit survey captured individual-level data including clinical, sociodemographic, and costing variables from 131 women in the study who tested positive for syphilis in Blantyre-area centers. Healthcare workers in ANC and sexually transmitted infection (STI) clinics of all participating health centers were invited to participate in semi-structured in-depth interviews (SSIs) which were conducted in Chichewa, translated to English, and then checked for accuracy. A topic guide based on the Every Newborn Action Plan essential health system components, adapted for prevention of mother-to-child-transmission (PMTCT) of syphilis, provided the framework for the SSIs.^8^

A National Clinic Readiness survey was conducted via a face-to-face interview with the staff member responsible for PMTCT services at 58 primary healthcare sites offering maternity care across the country. The survey assessed key coverage indicators related to supply chain and human resources.

Quantitative data were collected on REDCap-enabled electronic tablets and analyzed using Stata version 14.2. National Clinic Readiness data were summarized using descriptive statistics. Outcomes of interest were (1) availability of essential resources, (2) process of PMTCT service delivery, and (3) methods of routine documentation and surveillance. Data from the individual exit surveys at ANC1 and delivery were analyzed to (1) measure EMTCT process outcomes at participating health centers individually and by region and to (2) estimate the incidence of CS using adequate maternal treatment according to both the WHO surveillance definition (at least 1 dose of IM BPG >30 days before delivery) and the local clinical case diagnosis definition (3 doses of IM BPG >30 days before delivery). Data was used to inform two modified Tanahashi models. The *ANC model* assessed coverage among all recruited ANC1 attendees through three sequential stages: availability (women attending ANC <28 weeks), accessibility (women <28 weeks tested for syphilis), and effective coverage (seropositive women receiving same-day IM BPG). The *postnatal model* measured coverage through women recruited at delivery using four stages: availability (women tested at all during pregnancy), accessibility (seropositive women receiving ≥1 IM BPG dose in ANC), and the two effective coverage definitions—WHO (≥1 IM BPG dose >30 days before birth or infant treated) and local (3 IM BPG doses >30 days before birth or infant treated).

Individual-level costing analyses (n = 131) were conducted in Microsoft Excel (Microsoft Corporation, Redmond, WA). Socioeconomic measures (e.g., monthly income, livelihood, and perceived social status via a six-rung Economic Ladder Question^9^) and their out-of-pocket health expenditures (e.g., service costs, transport costs) were analyzed using descriptive statistics. Mean expenditures were calculated and stratified by livelihood type, facility type, transport mode, service type, and relative socioeconomic status. Missing cost data were excluded from the analysis for the corresponding variable. A catastrophic health expenditure threshold was defined as 40% of nonfood household consumption using Malawi’s Fifth Integrated Household Survey 2019 to 2020 data and used as a benchmark against which reported out-of-pocket costs were compared.^10^

Qualitative data were uploaded to NVivo following translation and transcription, and later in Taguette, to support data analysis. Qualitative data were first analyzed deductively against the stages of each Tanahashi model and the four pillars of the 2025 WHO Triple Elimination Framework to explore convergence with quantitative findings. A secondary inductive phase employed reflexive thematic analysis to identify broader themes that explained observed bottlenecks.^11^

## RESULTS

A staff member at each of the 58 facilities completed the National Clinic Readiness survey. 9513 pregnant women at ANC1 and 4724 at delivery completed the exit survey, and the 131 women who tested positive for syphilis completed a separate survey collecting sociodemographic and costing data within Blantyre-area centers. Twenty-nine qualitative SSIs were conducted.

### National Clinic Readiness Surveys

The clinic survey found that syphilis testing was not offered by ANC midwives in any facility (Table 1). Testing was typically conducted in a separate location (STI/outpatient department [OPD] or voluntary counseling and testing location). The survey also identified variation in data capture processes related to EMTCT of syphilis. While all clinics recorded maternal treatment in the STI clinic register, only 41/58 clinics (70.7%) recorded it in the maternal health passport, the document which would routinely be available at the point of delivery. We also identified two distinct approaches to the capture of maternal syphilis in the HMIS; 11/58 clinics (19%) counted each episode of treatment, whereas 39/58 clinics (62.7%) counted each woman with a positive test.

**TABLE 1.** Clinic Readiness to Provide EMTCT of Syphilis

Facility Demographics	N (%)
Health center region	
Southern	19 (32.8%)
Central	21 (36.2%)
Northern	18 (31%)
Sessions per week of ANC	
0	1 (1.7%)
1	0
2–5	42 (72.4%)
6–10	15 (25.9%)
ANC services	**N (%**)
Routine syphilis screening offered to pregnant women	
No	1 (1.7%)
Yes	57 (98.3%)
Method of syphilis testing offered	
Rapid point-of-care test (RPOCT) after finger prick	56 (98.2%)
Rapid plasma reagin (RPR) test	0
Venereal disease research laboratory (VDRL) test	0
2-step testing (RPOCT + RPR)	1 (1.7%)
No routine tests	1 (1.7%)
Location of syphilis testing	
ANC	0
Voluntary testing and counselling	57 (98.3%)
STI/OPD	1 (1.7%)
Is IM BPG offered onsite	
No	0
Yes	58 (100%)
Where is IM BPG administered	
In ANC on the same day	11 (19%)
OPD/STI clinic	47 (81.3%)
If treatment not available where is treatment advised	
Neighboring health center	7 (12.1%)
District hospital	5 (8.6%)
Private clinic	2 (3.5%)
Pharmacy and return for administration	44 (75.9%)
Offer partner notification	
No	2 (3.5%)
Yes	56 (96.6%)
Where are partners treated	
ANC	18 (31%)
STI	35 (60.3%)
Referral center	2 (3.5%)
Other	1 (1.7%)
No response	2 (3.5%)

Identification of CS at Birth	N (%)
Routine retesting at delivery No Yes Do not know	48 (82.8%) 7 (12.1%) 3 (5.2%)
Untested women tested at delivery	
No Yes Do not know	26 (44.8%) 29 (50%) 3 (5.2%)
Syphilis treatment status review at delivery No Yes Do not know	35 (60.3%) 20 (34.5%) 3 (5.17%)
Which infants receive treatment Asymptomatic infant/mother treated Asymptomatic infant/mother untreated Symptomatic infant Small/preterm infant/mother treated Do not know Other	1 (1.7%) 11 (19%) 44 (76%) 0 1 (1.7%) 1 (1.7%)
What treatments are offered to infants IM BPG Intravenous benzylpenicillin—10 days Referral to the district Never treated CS Other	40 (69%) 2 (3.4%) 6 (10.3%) 5 (8.6%) 5 (8.6%)

Surveillance and Data Capture	N (%)
How is clinical data captured at ANC Electronic medical record HMIS Maternal health passport Register filled by healthcare worker Combination Other	4 (6.9%) 44 (75.9%) 54 (93.1%) 48 (82.8%) 4 (6.9%) 1 (1.7%)
Where is maternal syphilis treatment captured ANC register Maternal health passport STI register	15 (25.9%) 41 (70.7%) 58 (100%)
If women buy the drug, where is it captured ANC register STI register Maternal health passport	23 (39.7%) 49 (84.5%) 48 (82.8%)
How is treatment captured in HMIS Each episode of treatment is counted Each woman treated is counted No response	11 (19%) 39 (67.2%) 8 (13.8%)
If an infant is diagnosed with CS where is it recorded Maternity register Postnatal ward register Maternal health passport Neonatal section of Maternal health passport Never treated CS STI/OPD/Neonatal register	18 (31%) 36 (62.1%) 29 (50%) 47 (81%) 3 (5.2%) 11 (19%)

While IM BPG was the standard of care at all facilities, it was only administered at ANC in 11/58 (18.9%) clinics. During IM BPG stock-outs, most clinics (44/58, 75.9%) advised women to procure the injection at a private pharmacy and return for administration. There was marked variation in approaches to the identification of infants at risk of CS at birth; 48/58 facilities (82.8%) did not routinely retest all patients for syphilis at delivery. Half of the clinics retested women lacking a testing history. Most, but not all facilities (44/58, 75.9%) treated symptomatic infants, but only 11/58 (19%) treated asymptomatic infants of untreated seropositive mothers, with only one clinic (1.7%) treating all asymptomatic infants born to seropositive mothers. Most (40/58, 69%) were able to treat infants with IM BPG; 6/58 clinics (10.9%) referred infants elsewhere.

**Figure 1.**
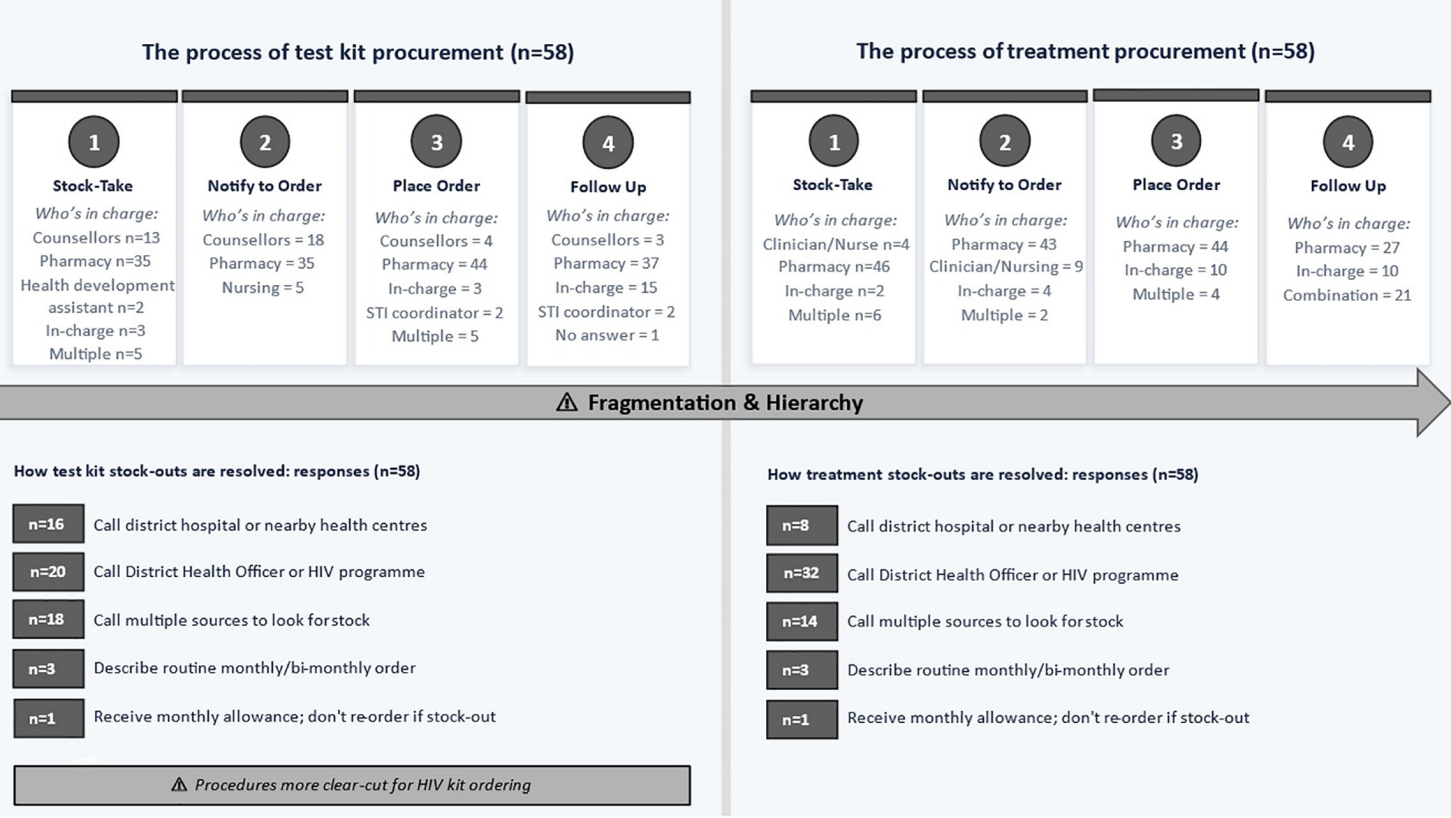
Syphilis test kits and treatment procurement process, roles, and stock-out actions according to National Clinic Readiness survey respondents.

At the end of the survey, participants were asked what they felt was the biggest barrier to EMTCT of syphilis at their facility. 41/58 (70.7%) respondents identified stock-outs of test kits and treatment as the most significant barrier to EMTCT of syphilis (Fig. 1).

### Regional Coverage of EMTCT Validation Outcomes

**Figure 2.**
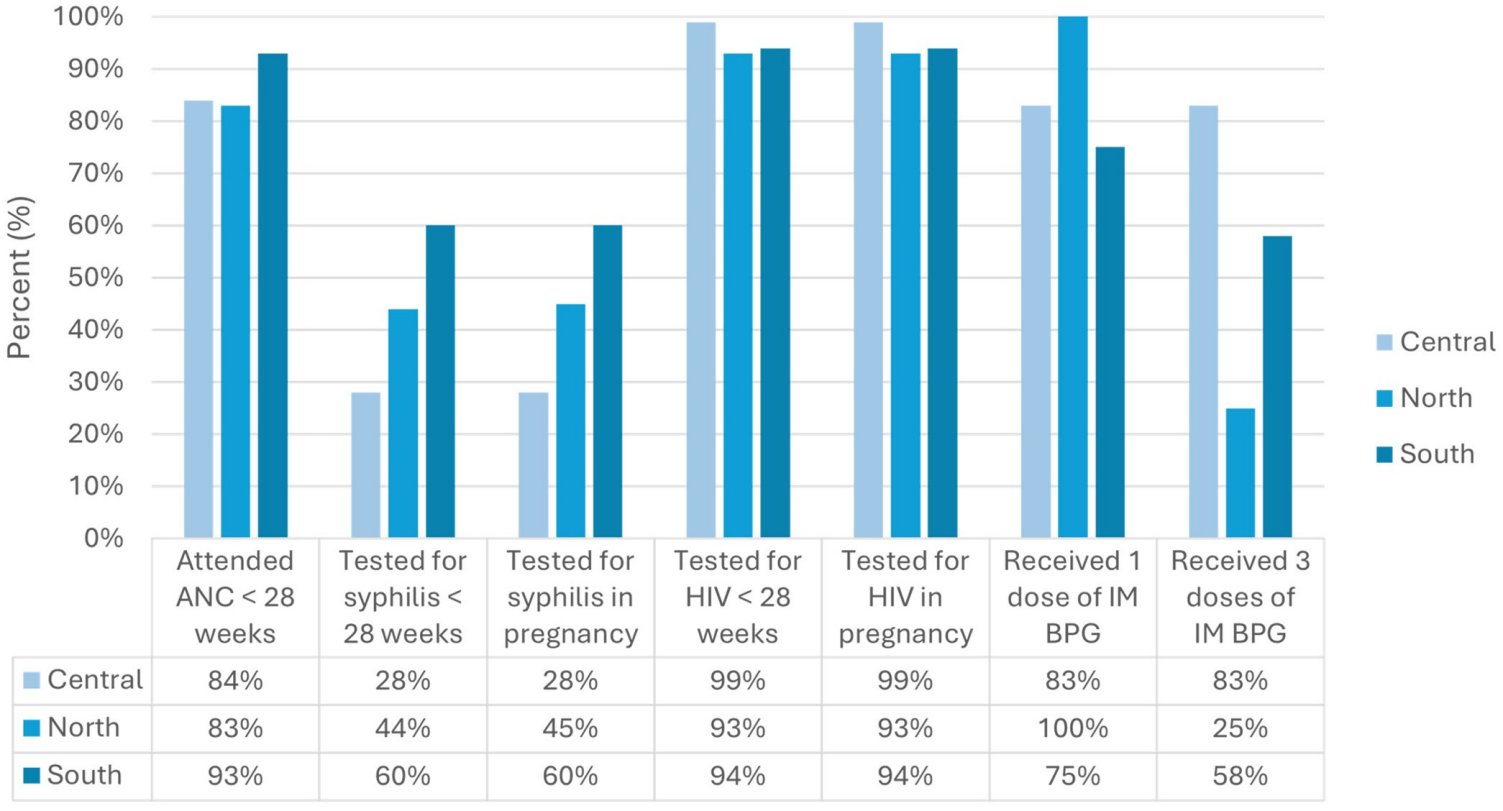
Coverage of WHO EMTCT of syphilis process outcomes by study region.

Individual data collected at ANC (n = 9513) and delivery (n = 4724) were analyzed to determine the coverage of (1) EMTCT process outcomes (Fig. 2) and (2) the incidence of CS, defined using both the WHO surveillance definition and the local clinical case definition (Supplemental Table S1, https://links.lww.com/OLQ/B367).

### Economic Attributes and Out-of-Pocket Expenditures

Individual-level cost-of-illness data were collected at the point of delivery from 131 women who tested positive for syphilis. 98/131 (74.8%) respondents relied on unpaid housework or farmwork for income. In the survey, 125/131 (95.4%) women responded to a six-rung Economic Ladder Question,^9^ of which 99 (79.2%) placed themselves in the bottom third. Of 81/131 (61.8%) women who responded to questions on service prices, 25 of these (30.8%) purchased their treatment. 74/131 (56.5%) women provided numerical data on out-of-pocket clinical and transport expenditures. Of these, 25/74 (33.8%) reported paying out-of-pocket for treatment costs, with a mean cost of 4344 Malawi kwacha (MWK). A further 9/74 (12.2%) paid for private consultation fees averaging MWK 4722, and 3/74 (4.1%) paid laboratory fees averaging MWK 3000. Using our findings, the average cost of a private consultation, lab fees, and treatment for one syphilis episode (12,066 MWK total) represents about 7% of the average catastrophic health expenditure threshold derived from national consumption data.^10^

### Bottlenecks in EMTCT Delivery Pathways

**Figure 3.**
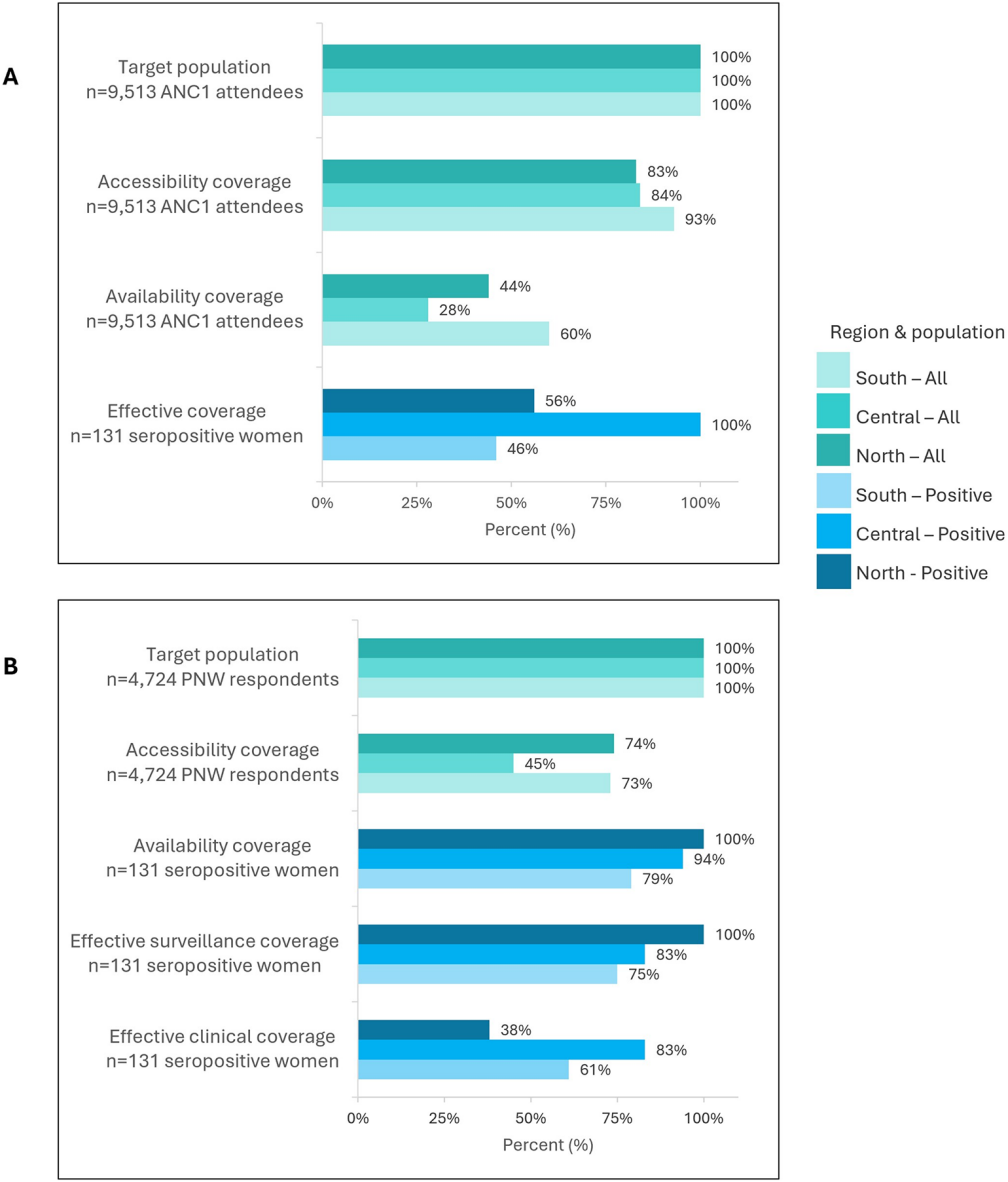
Modified Tanahashi models. (A) *ANC model* identifying bottlenecks to treatment at ANC per region. (B) *Postnatal model* identifying bottlenecks to CS PMTCT at delivery per region.

The individual exit surveys from ANC1 (n = 9513) were used to populate a Tanahashi model (*ANC model*) to describe the bottlenecks in the delivery of STAT at ANC. The bottlenecks were testing for syphilis <28 weeks (28%–60%), despite high ANC attendance <28 weeks (83%–94%), and at receipt of one IM BPG dose at ANC1 (46%–100%), which showed variability across regions (Fig. 3A). Coverage definitions for the model are available in Supplemental Table S2, https://links.lww.com/OLQ/B367.

The individual exit surveys from postpartum women (n = 4724) were used to populate a Tanahashi model (*postnatal model*) to evaluate bottlenecks in the provision of effective services to prevent or treat CS. Both clinical and surveillance coverage of CS were evaluated and are defined in Supplemental Table S2, https://links.lww.com/OLQ/B367. The bottlenecks in this model were poor coverage of maternal testing at ANC (45%–74%) and variable coverage of maternal treatment; between 79% and 100% of women received one IM BPG dose before delivery, but only 38% to 81% of women received three IM BPG doses (Fig. 3B).

### Qualitative Analyses

Twenty-nine health workers participated in SSIs across 6 sites; reported occupation titles are included in Supplemental Table S3, https://links.lww.com/OLQ/B367.

### ANC Attendance

Respondents confirmed high uptake of ANC services. Women were described as intrinsically motivated to protect their baby’s health: “*I do not think such a woman can refuse. This is because it is every woman’s dream to give birth to a healthy baby*” (Health Worker, Mpemba).

High acceptance of ANC screening services was attributed to routine health talks and implicit opt-out screening standards. Respondents also credited the role of community sensitization by traditional chiefs and radio campaigns:

*“Chiefs in the villages explain to pregnant women about the importance of starting [ANC] at early stage …. When other women hear that information, it makes them to start attending [ANC] at an early stage so that they can be assisted quickly.” Health Worker, Karonga*

### Testing Bottlenecks

Test kit stock-outs were the predominant barrier to PMTCT of syphilis, with providers reporting months-long gaps: “*[S]yphilis test kits do not last long … as we are talking now, we do not have these test kits so the women that are coming for antenatal now, are just passing without a syphilis test*” (Health Worker, Mpemba). Retesting at delivery, mandated by local guidelines, was rarely practiced due to kit shortages or prioritization of Human Immunodeficiency Virus (HIV) testing: “*We don’t test during labor … most of the times we don’t test syphilis on these women, we test the HIV … sometimes we have no kits*” (Health Worker, South Lunzu). Maternal health passports served as the primary testing record, but could be prone to oversights and resulting missed EMTCT opportunities: “*Sometimes we discover … a positive result documented but was overlooked*” (Health Worker, Chilumba).

### Treatment Bottlenecks

IM BPG shortages regularly constrained treatment coverage. When stock was available, respondents indicated that syphilis treatment is deprioritized in favor of other conditions, forcing patients to purchase doses out-of-pocket:

*“… [T]reatment, it’s a huge problem. We receive a small consignment of benzathine, so most of the times [sic], people with STIs are the least to be considered. They first give to people with heart problems, sickle cells and other conditions … Thereafter, we just advise them to buy.” Health Worker, Chilumba*

Respondents also noted clinical congestion as a barrier to effective treatment:

*“… [W]e have a congested environment … when positive, we send her back to antenatal where they also sent her to meet the doctor… [then] sent to the pharmacy for the treatment, and then … to the doctor to get administered.” Health Worker, South Lunzu*

### Service Integration and Disparities

HIV services consistently outranked syphilis in training, supply priority, and community outreach: “*[Syphilis is] a disease that [we] look down on, we focus more on HIV*” (Health Worker, Bangwe). Many reported receiving training for HIV, but most had received no syphilis-specific training. A health worker in South Lunzu summarized the disparity by simply stating “… *the HIV drugs are always available*.”

## CONCLUSIONS

Our assessment of EMTCT services in Malawi identified the primary barrier to care delivery as the frequent and widespread stock-outs of test kits. Qualitative data confirmed that stock-outs arose due to issues with ordering and supply chain at the clinic level, as well as funding fragmentation and prioritization of HIV at national and clinic levels. Despite these challenges, we identified several supportive factors, including high coverage of ANC attendance and high testing uptake driven by intrinsic motivation, in-clinic messaging, and positive community influences. Our findings align with evidence from Indonesia describing more frequent and prolonged syphilis and hepatitis B test stock-outs than HIV.^12^ They are also consistent with a 2025 analysis from Zambia, which identified low syphilis testing as a consequence of supply chain failures rather than a lack of patient demand.^13^ Options to improve testing coverage include integrating syphilis testing into HIV supply chains and deploying affordable dual HIV-syphilis rapid tests (currently treponemal-only).^14,15^ The advent of newer point-of-care immunoglobulin M and nontreponemal tests may provide additional options in future.^16^

None of the sites offered testing directly within ANC, forcing patients to navigate congested health facility environments to access services. This care fragmentation likely contributed to the identified testing bottleneck and presented potential missed opportunities for EMTCT of syphilis. Existing evidence confirms that syphilis screening in ANC clinics is feasible, a practice explicitly supported by WHO.^17,18^ An analysis of countries that have achieved HIV and syphilis EMTCT validation reveals that routine antenatal syphilis screening is a key attribute of their success. Future research should investigate the barriers behind testing pathway disruptions, as addressing these transitions is critical to improving screening coverage.

Along with challenges with testing, we found IM BPG supply was interrupted at local, national, and international levels, which has been demonstrated in both national and international analyses from other regions.^19,20^ These stock-outs compelled most facilities to ask women to source IM BPG privately, though our data did not capture whether women returned for administration. Consequently, our study likely underestimates the true gap in effective treatment. While the logistics of IM BPG stock-outs are well documented, the qualitative data from this study revealed that health centers may prioritize available stock for other illnesses. In contrast, one ANC in-charge officer reported maintaining a dedicated stock for pregnant women at ANC, highlighting the importance of local leadership and advocacy. These findings underscore the value of mixed-method analysis in the evaluation of EMTCT services.

Buying IM BPG privately during stock-outs resulted in mothers paying out-of-pocket costs. Although the costs fell below the catastrophic threshold for the average Malawian household, they may still impose a substantial financial burden on poorer households whose nonfood consumption is well below the national mean.^10,21^ As cost data were collected from only 56.5% of syphilis-positive respondents, and were limited to peri-urban and urban sites in Blantyre, the findings may reflect an upper-bound for service prices, yet underestimate the true financial burden on rural households. To our knowledge, this is the first study to report patient-associated healthcare costs for antenatal syphilis management. Given that many women will test positive on treponemal point-of-care tests in consecutive pregnancies, as these tests remain positive for life, repeat out-of-pocket expenditure, which may not be necessary, is essential to understand.

Previous analysis of Malawian HMIS data identified variable data capture methods for gestational syphilis as a barrier to accurate estimation of prevalence.^6^ Our National Clinic Readiness survey identified variability in individual and surveillance level data capture; documenting this will support the design of interventions to support standardization.^22,23^ Identification of infants with CS at birth, which is essential for both clinical care and surveillance, was particularly poor. In high-income settings, diagnosis of CS relies on complex diagnostic algorithms, whereas in resource-limited settings, treatment is given to infants “at risk” of CS without a confirmed diagnosis.^24^ Our data showed inconsistent practices and missed opportunities for identification of CS at birth in Malawi, including incomplete maternal treatment records, lack of postnatal systems to identify at-risk infants, and limited neonatal treatment capacity at the primary care level. The issues with CS surveillance are illustrated by a marked discrepancy between the estimated incidence based on the local clinical case definition of at-risk infants and the WHO surveillance definition (Supplemental Table S1, https://links.lww.com/OLQ/B367). The true burden of CS remains unknown due to limitations in routine testing strategies and data collection. Strengthening clinical and surveillance data capture at antenatal and postnatal levels will be key to improving case linkage, enhancing surveillance accuracy, and advancing Malawi’s progress toward EMTCT targets.

A secondary output of this study was to pilot a suite of data collection and analysis tools that could be used for longitudinal assessment of Malawi’s progress toward EMTCT of syphilis, or adapted for use in other settings (Supplemental Toolkit 1, https://links.lww.com/OLQ/B382 and Supplemental Toolkit 2, https://links.lww.com/OLQ/B383). The National Clinic Readiness survey proved to be a rapid, low-cost, high-value instrument that provided critical context related to local supply chain constraints. Individual-level exit surveys were similarly low-cost and captured regional variation in service delivery. However, future implementation would benefit from standardized, regionally representative site selection, such as fewer surveys per site or a point-prevalence design, to address the missing rural data in the Southern and Central regions that likely introduced biases into these coverage estimates. As site selection was tied to existing research sites, generalizability of findings at a national level or transferability to other country settings is limited. The Tanahashi models effectively quantified regional bottlenecks along the EMTCT pathway but, depending on local priorities, evaluating same-day testing and treatment at ANC or identification of CS could be prioritized. The separate patient-level survey for seropositive study participants, while resource-intensive, generated patient-reported estimates of out-of-pocket costs. Nonresponse was common for cost-related questions, particularly those requiring numerical recall of costs. Clearer definitions of key terms and structured response formats (e.g., banded ranges or multiple-choice options) could improve completeness and reduce recall bias.

The qualitative component provided critical context and revealed facility-level dynamics underpinning many quantitative findings. While our qualitative approach captured initial insights into dynamics between pregnant women and their partners, future iterations of this toolkit should incorporate more comprehensive partner-related data collection and analysis. Given the central role of partner testing and treatment in syphilis prevention and reinfection, inclusion is critical for a comprehensive understanding of the care pathway. The 2025 WHO-UNICEF Triple Elimination guidance, centered on four pillars, may also replace the Every Newborn Action Plan framework in future adaptations of these qualitative instruments.^1^ Collectively, our proposed toolkit integrates clinical, surveillance, and system-level data with health worker perspectives, providing a granular view of a country’s EMTCT status and identifying barriers often missed by routine system-level data.

Malawi has demonstrated a strong political commitment to addressing EMTCT of syphilis alongside HIV through integrated service delivery approaches.^25^ Despite high demand for ANC, syphilis testing, and treatment health system bottlenecks persist that may impede achievement of the WHO EMTCT validation criteria. Findings from our study may aid in prioritizing and monitoring future initiatives supporting EMTCT and the broader Triple Elimination agenda.

## Supplementary Material
