# Supplementary material for: The Road to Elimination of Mother-to-Child Transmission of Syphilis in Malawi: A Mixed-Methods Analysis of Health System Readiness
Source: Sex Transm Dis. 2026 May 18;53(8):492–9. doi: 10.1097/OLQ.0000000000002359 (PMC13326934; doi:10.1097/OLQ.0000000000002359)
Supplement: Supplementary file 1 [file std-53-492-s001.pdf]

***The road to EMTCT of syphilis in Malawi: a mixed-methods analysis of health system readiness*: Supplemental Tables, Tools, and Files**

Part 1: Supplementary Data Tables

Table S1: Incidence of congenital syphilis in Malawi*

	WHO CS definition	Malawi CS definition
Total	1,223.34	1,976.16
Region		
Central	738.92	738.92
North	0	751.88
South	1,815.43	2,723.15
Location		
Rural	0	1,593.63
Urban	2,129.31	3,246.43

* Denominators included health passport extraction at the postnatal ward, which did not exclude stillbirths, but also may have missed women who delivered stillborn infants and who went home directly from the delivery suite.

Table S2: Coverage definitions of each of the two Tanahashi models

**ANC Tanahashi Model**	
**Target Population**	All women recruited at ANC
**Availability Coverage**	Women attending ANC <28 weeks
**Accessibility Coverage**	Women attending ANC <28 weeks and tested for syphilis
**Effective Coverage**	Seropositive women who receive one IM BPG dose on the same day
**Post-natal Tanahashi Model**	
**Target Population**	All women recruited at delivery
**Availability Coverage**	Women tested at any stage during pregnancy
**Accessibility Coverage**	Seropositive women receiving at least one IM BPG dose during the ANC period
**Effective Coverage** (WHO surveillance definition)	Seropositive women receiving one IM BPG dose >30 days before birth, or infant treated if maternal treatment inadequate
**Effective Coverage** (Malawi clinical definition)	Seropositive women receiving three IM BPG doses >30 days before birth, or infant treated if maternal treatment inadequate

Table S3: Reported occupation titles of SSI respondents

Title	N
Nurse/Midwife	5
Midwife	5
Nurse/Midwife technician	2
Nursing in-charge	2
Nursing officer	1
Clinician	3
Clinical officer	1
Clinical medical assistant	1
Medical assistant	1
HIV diagnostic assistant	5
HIV testing services counselor	1
Health surveillance assistant (HSA)	1
Not recorded	1

Part 2: Data collection & analysis tools
For additional information on access to and implementation of these tools, please contact the study team directly.

Index:

1. **Supplemental Toolkit 1**
    a. National Clinic Readiness Survey
    b. ANC Subsequent Exit Questionnaire
    c. Postnatal Exit Questionnaire
    d. Socioeconomic & Costing Exit Questionnaire
    e. Health Care Worker SSI Guide
    f. Pregnant women SSI Guide
2. **Supplemental Toolkit 2: EMTCT quantitative analysis files (R files)**
