# Supplementary material for: The Road to Elimination of Mother-to-Child Transmission of Syphilis in Malawi: A Mixed-Methods Analysis of Health System Readiness
Source: Sex Transm Dis. 2026 May 18;53(8):492–9. doi: 10.1097/OLQ.0000000000002359 (PMC13326934; doi:10.1097/OLQ.0000000000002359)
Supplement: Supplementary file 2 [file std-53-492-s002.pdf]

National Clinic Readiness Survey

Variable Name	Question Text	Saved Value	
_qdate_sys	Today's date	User entered text	
bas_consent	Has the participant given a verbal consent?	User entered text	
Thank you for agreeing to participate in this questionnaire. We are trying to assess implementation gaps in the delivery of PMTCT of syphilis in Malawi. We are not assessing the performance of your particular health facility and all of the information yougive will be kept confidentially and not shared in association with the name or location of your facility or your name. Please answer the following questions freely. Please don't answer what you think "should" happen or is "recommended" to happen but what you believe happens on the ground. If you are not sure because your role doesn't permit you to have that on the ground experience please just indicate you are not sure. If this happens several times we may ask if there is someone else at your facility who is better placed to answer. Thanks again for your help and your commitment to quality improvement in this area.			
bas_hfname	1. Health facility name	User entered text	
bas_title	1a. Official job title	User entered text	
bas_years	1b. Number of years in that position at that health facility	User entered integer	
	2. What day(s) does ANC take place at this facility:		
bas_monanac	Monday	1	AM
		2	PM
		3	Both AM and PM
		4	Unable to answer this question as it is outside my scope of practice / experience
		0	None
bas_tueanc	Tuesday	1	AM
		2	PM
		3	Both AM and PM
		4	Unable to answer this question as it is outside my scope of practice / experience
		0	None
bas_wedanc	Wednesday	1	AM
		2	PM
		3	Both AM and PM
		4	Unable to answer this question as it is outside my scope of practice / experience
		0	None
bas_thuranc	Thursday	1	AM
		2	PM
		3	Both AM and PM

		4	Unable to answer this question as it is outside my scope of practice / experience
		0	None
bas_frianc	Friday	1	AM
		2	PM
		3	Both AM and PM
		4	Unable to answer this question as it is outside my scope of practice / experience
		0	None
bas_syphillis	3. Is syphilis testing offered as part of routine ANC services at this site?	1	Yes
		0	None
		2	Syphilis testing is not routinely offered at this facility
bas_vdrl	3a. Laboratory based VDRL following venepuncture	1	Yes
		0	None
		2	Unable to answer this question as it is outside my scope of practice / experience
bas_rpr	3b. Laboratory based RPR following venepuncture	1	Yes
		0	None
		2	Unable to answer this question as it is outside my scope of practice / experience
bas_rpoct	3c. Rapid POCT following venepuncture	1	Yes
		0	None
		2	Unable to answer this question as it is outside my scope of practice / experience
bas_prick	3d. Rapid POCT following finger prick	1	Yes
		0	None
		2	Unable to answer this question as it is outside my scope of practice / experience
bas_vdrlrpr	3e. A combination of laboratory based VDRL or RPR in parallel with rapid POCT (i.e. both types of test done on each patient)	1	Yes
		0	None
		2	Unable to answer this question as it is outside my scope of practice / experience
bas_rprpoct	3f. A combination of laboratory based VDRL or RPR and rapid POCT depending on availability of tests	1	Yes
		0	None
		2	Unable to answer this question as it is outside my scope of practice / experience

bas_vdrlother	3g. Other	1	Yes
		0	None
		2	Unable to answer this question as it is outside my scope of practice / experience
bas_vdrl_spec	3h. Other syphillis testing strategy	User entered text	
bas_syphtest	4. Where is syphilis testing offered in this HC?	1	By midwife / clinician in ANC
		2	By counsellor in VTC centre
		3	In STI clinic / OPD
		4	Syphilis testing is not routinely offered at this facility
		5	Unable to answer this question as it is outside my scope of practice / experience
		9	other
bas_testother	4a. Where else is testing offered?	User entered text	
bas_syphpreg	5. When Benzathine Penicillin is available, is syphilis treatment offered at this site?	1	Yes
		0	None
		2	Unable to answer this question as it is outside my scope of practice / experience
bas_rxwhere	5a. Where is it administered?	1	In the ANC on the time of diagnosis
		2	At ANC on a different day
		3	At OPD / STI clinic
		4	Unable to answer this question as it is outside my scope of practice / experience
		8	Other
bas_stiopen	5b. Is OPD/STI clinic ALWAYS open at same times as ANC clinic?	1	
		0	
bas_wherespec	5c. Where else is treatment administered?	User entered text	
bas_rxno	6. If treatment is not offered at this site where are test-positive women referred?	1	Neighbouring HC
		2	District hospital
		3	Tertiary/ Central hospital
		4	Private Clinic
		5	Advised to buy privately and return
		6	Unable to answer
		7	Other
bas_rxnospec	6a. Where else are test-positive women referred	User entered text	
bas_rxyes	6b. If treatment is offered but not available where are test-positive women referred?	1	Neighbouring HC
		2	District hospital
		3	Tertiary/ Central hospital
		4	Private Clinic
		5	Advised to buy privately and return

		6	Unable to answer
		7	Other
bas_rxelse	6c. Where else are test-positive women referred?	User entered text	
bas_kitsstock	7. Who is responsible for: Stock take (identifying when kits are low)	User entered text	
bas_newkits	7a. Notifying need to order new kits	User entered text	
bas_order	7b. Placing order for new kits	User entered text	
bas_follow	7c. Following up on order	User entered text	
bas_kitsfrom	7d. Where do you order syphilis test kits from?	User entered text	
bas_kprocess	7e. What is the process for replacing kits when you have a stock out? (record verbatim the text)	User entered text	
bas_kitqc	7f. What is the process for test kit quality control and validation on receiving a new batch?	User entered text	
bas_kitres	7g. Who is responsible for test kit quality control and validation on receiving a new batch?	User entered text	
	8. What day(s) does treatment take place at this facility:		
bas_monrx	Monday	1	AM
		2	PM
		3	Both AM and PM
		4	Unable to answer this question as it is outside my scope of practice / experience
		0	None
bas_tuerx	Tuesday	1	AM
		2	PM
		3	Both AM and PM
		4	Unable to answer this question as it is outside my scope of practice / experience
		0	None
bas_wedrx	Wednesday	1	AM
		2	PM
		3	Both AM and PM
		4	Unable to answer this question as it is outside my scope of practice / experience
		0	None
bas_thurrx	Thursday	1	AM
		2	PM
		3	Both AM and PM
		4	Unable to answer this question as it is outside my scope of practice / experience

		0	None
bas_frirx	Friday	1	AM
		2	PM
		3	Both AM and PM
		4	Unable to answer this question as it is outside my scope of practice / experience
		0	None
bas_meds	9. Who is responsible for: Stock take (identifying when medication is low)	User entered text	
bas_newmeds	9a. Notifying need to order new medication	User entered text	
bas_ordenew	9b. Placing order for new medication	User entered text	
bas_fuporder	9c. Following up on order	User entered text	
bas_repbenza	9d. Where do you order syphilis medication from?	User entered text	
	9e. What is the process for replacing medication when you have a stock out? (record verbatim the text)	User entered text	
bas_partner	10. Is partner notification routinely offered?	1	Yes
		0	None
		2	Unable to answer this question as it is outside my scope of practice / experience
bas_rxpart	10a. Where are partners advised to attend for treatment?	1	Yes
		0	None
		2	Unable to answer this question as it is outside my scope of practice / experience
bas_rxpartelse	10b. Where else are partners advised to attend for treatment?		
bas_syphdel	11. Are all women routinely retested for syphilis at the time of delivery?	1	Yes
		0	None
		2	Unable to answer this question as it is outside my scope of practice / experience
bas_syphretest	12. Are women retested for syphilis at the time of delivery if they have not previously been tested in that pregnancy?	1	Yes
		0	None
		2	Unable to answer this question as it is outside my scope of practice / experience
bas_rxteview	12a. Prior to discharge are a woman's syphilis status and treatment history reviewed on the	1	Yes
		0	None

	postnatal ward?	2	Unable to answer this question as it is outside my scope of practice / experience
bas_congesyph	13. Which infants are identified as needing treatment for congenital syphilis? In these questions a "syphilis exposed" infant is one whose mother had a positive syphilis test in pregnancy	1	A syphilis exposed infant who has no
		2	A syphilis exposed infant who has no
		3	A syphilis exposed infant who has signs of congenital syphilis at birth (jaundice, rash)
		4	A syphilis exposed infant who is preterm or low birth weight but otherwise asymptomatic and the mother has received ALL her treatment in pregnancy.
		6	Unable to answer this question as it is outside my scope of practice / experience
		8	Other

ANC Subsequent Exit Questionnaire

Variable Name	Question Text	Saved Value	
_qdate_sys	Today's date	User selected date	
anc_f_id	Health facility ID	User entered text	
pid_valid	Enter a valid Participant ID	User entered text	
anc_ancvisit	1. Which ANC visit did this woman have today?	1	ANC1
		2	ANC2
		3	ANC3
		0	ANC4
		8	ANC5+
anc_anc1av	1a. Is the date of ANC1 available?	1	Yes
		0	No
anc_anc1dt	1b. Date of ANC1	User selected date	
anc_gavail	2. Is the gestational age of this pregnancy today available?	1	Yes, weeks only
		2	Yes, days only
		3	Yes, both weeks and days
		9	No, not documented
anc_gestationw	2a. Gestational age in weeks:	User selected integer	
anc_gestationd	2b. Gestational age in days:	User selected integer	
anc_syphdoc	3. Does this woman have a documented syphilis test results in her passport from a previous ANC visit?	1	Yes
		0	No
anc_pos	3a. You indicated she was previously tested – was the test positive?	1	Yes
		0	No
anc_dtprev	3b. Is the date of positive test available?	1	Yes, Complete (Day-Month-Year)
		2	Yes, Partially complete (Month-Year)
		3	Yes, Partially complete (Year Only)
		99	Date not documented
anc_prevpos	3c. Date of positive test	User selected date	
anc_preposm	3d. Month & year of positive test	User selected date	
anc_preposyr	3e. Year of positive test	User selected date	
anc_syphtest	4. You indicated she previously tested positive, has she received treatment yet?	1	Yes
		0	No
anc_doses	4a. How many doses?	1	1
		2	2
		3	3
		8	Other
anc_inj1doc	4b. Was the first dose documented?	1	Yes
		0	No
anc_inj1dt	4c. Date of first injection	User selected date	
anc_inj2doc	4d. Was the second dose documented?	1	Yes
		0	No
anc_inj2dt	4e. Date of second injection	User selected date	
anc_inj3doc	4f. Was the third dose documented?	1	Yes
		0	No

anc_inj3dt	4g. Date of third injection	User selected date	
anc_testtod	5. You indicated she was not previously tested, was she tested today?	1	Yes
		2	No
anc_notavail	5a. Why not?	1	Tests not offered
		2	Tests not available
		3	Staff not available
		4	Testing refused
		9	Other
anc_availspec	5b. Other reason:	User entered text	
anc_result	6. If she tested positive today, what was the test result?	1	Positive
		0	Negative
anc_rxtoday	6a. You indicated she tested positive for the first time today, did she receive the first dose of treatment today?	1	Yes
		0	No
For the next question, a negative HIV test prior to ANC 1 is not adequate and in this case the answer to this question = No. All women should have a HIV test repeated at ANC 1 unless they are known HIV positive. If they are known HIV positive prior to pregnancy the answer to this question =Yes			
anc_hprec	7. Does this woman have a documented HIV serostatus during this pregnancy?	1	Yes
		0	No
anc_sero	7a. What is her serostatus?	1	HIV Positive
		0	HIV Negative
anc_onartrx	7b. Has she started ART	1	Yes
		0	No
anc_rxartstart	7c. When did she start ART?	1	Pre-pregnancy
		2	Current pregnancy
		9	Not known

Postnatal Exit Questionnaire

Variable Name	Question Text	Saved Value	
_qdate_sys	Today's date	User selected date	
del_f_id	Health facility ID	User entered text	
pid_valid	Enter a valid Participant ID	User entered text	
del_gavail	1. Is the gestational age of this baby available:	1	Yes, Weeks only
		2	Yes, Days only
		3	Yes, Both Weeks and Days
		9	No, Not documented
del_gestationw	1a. What is the estimated gestation of the baby born today? Weeks:	User entered integer	
del_gestationd	1b. What is the estimated gestation of the baby born today? Days:	User entered integer	
del_live	2. Live born	1	Yes
		0	No
del_fsb	2a. Fresh still birth (FSB)	1	Yes
		0	No
del_premmie	2b. Preterm	1	Yes
		0	No
del_lbw	2c. Low birth weight (LBW) infant	1	Yes
		0	No
del_msb	2d. Macerated stillbirth (MSB)	1	Yes
		0	No
del_syphilis	3. Does this woman have a documented antenatal syphilis test result?	1	Yes
		0	No
del_docdate	3a. What date was it done on?	User selected date	
del_ancvisit	3b. What ANC visit was this at?	1	ANC1
		2	ANC2
		3	ANC3
		4	ANC4
		5	ANC5
		6	ANC5+
del_result	3c. What is the test result?	1	Positive
		0	Negative
del_treatment	4. Since test is positive, is there treatment documented during pregnancy?	1	Yes
		0	No
		9	Don't know
del_injections	4a. According to the health passport	1	1

	how many doses of treatment has the woman received?	2	2
		3	3
		9	Don't know
del_inj1doc	4b. Was the first dose documented?	1	Yes
		0	No
del_inj1dt	4c. Date of first injection	User selected date	
del_inj2dt	4d. Was the second dose documented?	1	Yes
		0	No
del_inj2dt	4e. Date of second injection	User selected date	
del_inj3doc	4f. Was the third dose documented?	1	Yes
		0	No
del_inj3dt	4g. Date of third injection	User selected date	
del_testtoday	5. Has this woman had a syphilis test today?	1	Yes
		0	No
del_restoday	5a. What is today's test result?	1	Positive
		0	Negative
del_recommend	6. Has infant treatment and follow-up been recommended?	1	Yes
		0	No
Note: Please refer to infant management SOP and discuss with managing clinical team as appropriate			

Socioeconomic & Costing Exit Questionnaire

Variable Name	Question Text	Saved Value	
_qdate_sys	Today's date	User selected date	
pos_f_id	Health facility ID	User entered text	
pid_valid	Enter a valid Participant ID	User entered text	
pos_status	1. Marital status?	1	Married
		2	Separated
		3	Co-habiting
		4	Divorced
		5	Widowed
		6	Single
pos_adults	1a. How many adults are in her household?	User entered integer	
pos_child	1b. How many children are in her household?	User entered integer	
pos_dob	2. Is the mother's DOB known:	1	Yes, Complete (Day-Month-Year)
		2	Yes, Partially complete (Month-Year)
		3	Yes, Partially complete (Year Only)
		99	Date not documented
pos_dob1	2a. What is the Mother's DOB	User selected date	
pos_educ	3. What is the mother's highest level of education?	1	No Formal Schooling
		2	Incomplete Primary
		3	Complete Primary School
		4	Incomplete Secondary School
		5	Complete Secondary
		6	Diploma
		7	University Degree
		8	Post graduate
		99	Declined to Answer
pos_ownincom	3a. Do you have a source of monetary income apart from that earned by your partner or parents?	1	Yes
		2	No
**I am going to ask you questions about items your household owns as part of this study. The study will not be giving out any household items to anyone participant in the study.**			
pos_elec	4. Does your household have electricity	1	Yes
		2	No
pos_radio	4a. Does your household have a radio	1	Yes
		2	No
pos_tv	4b. Does your household have a televison	1	Yes
		2	No
pos_bedmat	4c. Does your household have a bed with a mattress	1	Yes
		2	No

pos_sofa	4d. Does your household have a sofaset	1	Yes
		2	No
pos_mphone	4e. Does any member of this household own: a mobile phone?	1	Yes
		2	No
pos_bankacc	4f. Does any member of this household have a bank account?	1	Yes
		2	No
pos_matfloor	4g. What is the main material of the floor in your household?	1	Earth/Sand
		2	Cement
		8	Other floor material
pos_floorspec	4h. Other floor material	User entered text	
pos_matroof	4i. What is the main material of the roof in your household?	1	Thatch
		2	Palm leaf
		3	Metal
pos_mfuel	4j. What type of fuel does your household mainly use for cooking?	1	Wood
		8	Other fuel
	4k. Other fuel for cooking	User entered text	
pos_foodenuf	4l. In the past 7 days, did you worry that your household would not have enough food?	1	Yes
		2	No
pos_clothing	4m. Concerning your household's clothing, which of the following is true?	1	It was less than adequate for household needs
		2	It was just adequate for household needs
		3	It was more than adequate for household needs
**Show picture of steps to the participant**			
pos_step	5. Imagine six steps, where on the bottom , first step, stand poorest people and on thehighest step, the sixth, stand rich people. On which step are you today?	1	Step 1
		2	Step 2
		3	Step 3
		4	Step 4
		5	Step 5
		6	Step 6
pos_syphanc	6. At which ANC visit was she diagnosed with syphilis?	1	1
		2	2
		3	3
		4	4
		8	Other
pos_syphtreatdc	7. Did she receive treatment for syphilis in this pregnancy?	1	Yes
		2	No
pos_visitdos	7a. At which ANC visit did she receive her first dose of treatment	1	1
		2	2
		3	3
		4	4

		5	Attended for an additional visit to receive treatment
		8	Other
pos_syphtreatnd	7b. If not, why not?	1	Not offered treatment
		2	Allergic to penicillin
		3	Penicillin not available
		4	Treatment refused
		8	Other
pos_allerg	7c. If "allergic to penicillin" or "penicillin not available" was she referred to another facility?	1	Yes
		2	No
pos_treatddnt	7d. If referred, why did she not attend for treatment?	1	Couldn't afford attendance at referral
		2	Didn't trust care at referral facility
		3	Didn't have time for travel to referral facility
		4	Didn't want to have
		8	Other
pos_treatrec	7e. Where did she receive treatment?	1	ANC clinic (treatment
		2	ANC clinic (treatment purchased)
		3	STI clinic at same HC (treatment provided)
		4	STI clinic at same HC (treatment purchased)
		5	STI clinic at other HC (treatment provided)
		6	STI clinic at other HC (treatment purchased)
		7	STI clinic district or referral hospital
		8	STI clinic district or referral hospital
		9	Private clinic
		10	Other
pos_dose	7f. How many doses of treatment did she receive?	1	1
		2	2
		3	3
		9	Don't know
**Cost of illness**			
pos_travelfac	8. How did the woman travel to the facility?	1	Walked
		2	Bicycle
		3	Minibus/Taxi

		4	Motorcycle
		5	Personal car
pos_travelcst	9. How much did it cost her to travel from your home to this facility?	User entered integer	
pos_timespnt	10. How much time did she spend at the facility in order to access syphilis testing and treatment?	User entered integer	
pos_mainactvy	11. What would you otherwise have been doing as her main activity if you had not had to attend this facility today?	1	Housework / farmwork
		2	Voluntary work
		3	Leisure activities
		4	Attending school or
		5	Paid work
		6	Self employed work
pos_paidwrk	12. How many hours does she work in a typical	User entered integer	
pos_month	13. How much money does she make in a month	User entered integer	
pos_timeoff	14. If you took time off from paid work (or business activity if self-employed) to come to the facility today approximately how much time did they take off work (or business activity if self-employed)?	User entered integer	
pos_earnlost	14a. Did she lose earnings as a result?	1	Yes
		2	No
pos_recall	14b. Can she recall how much she lost?	1	Yes
		2	No
pos_losthw	14c. How much did she lose?	User entered integer	
pos_paybhalf	15. Did she have to pay someone to do work on her behalf?	1	Yes
		2	No
pos_paid	15a. Can she recall how much she paid?	1	Yes
		2	No
pos_payhw	15b. How much did she pay them?	User entered integer	
pos_payconst	16. Did she pay any consultation fees relating to syphilis?	1	Yes
		2	No
		9	Other
pos_payconhw	16a. How much did she pay?	User entered integer	
pos_paylab	17. Did she pay any laboratory costs?	1	Yes
		2	No
		9	Other
pos_paylabhw	17a. How much did she pay?	User entered integer	
pos_paydrug	18. Did she pay any fees for treatment or drugs?	1	Yes
		2	No
		9	Other
pos_paydrughw	18a. How much did she pay?	User entered integer	
pos_folowup	19. With regards to syphilis treatment only, is she required to make a follow up visit for her and the	1	Yes
		2	No
pos_folowhw	19a. How many follow up visits will she make?	User entered integer	

pos_syphtyms	20. How many times has she been here before for syphilis treatment?	User entered integer	
pos_outanc	20a. How many of these follow-up visits were outside her routine ANC appointments?	User entered integer	
**Companion costs**			
**For question 21, please make it clear this should only be if someone accompanied her specifically because of the diagnosis of syphilis, i.e. her partner for testing or treatment OR any other support person because she wanted emotional support or help in understanding her diagnosis from a**			
pos_escort	21. Did anyone accompany you today or any other day because of your diagnosis of syphilis?	1	Yes
		2	No
pos_escortpreg	21a. How many times?	User entered integer	
pos_whescort	21b. Who escorted the woman?	1	Spouse / sexual partner
		2	Relative
		3	Friend
		8	Other
pos_hhmembr	21c. Is this person a member of her household?	1	Yes
		2	No
pos_travelcom	22. How did they travel to the facility?	1	Walked
		2	Bicycle
		3	Minibus/Taxi
		4	Motorcycle
		5	Personal car
pos_hwcom	22a. How much did it cost them to travel from their home to this facility?	User entered integer	
pos_timechrs	24. How many hours did they spend at facility while waiting for you to access syphilis testing and	User entered integer	
pos_mainactvyc	25. What would they otherwise have been doing as their main activity if they had not come to this facility?	1	Housework / farmwork
		2	Voluntary work
		3	Leisure activities
		4	Attending school or
		5	Paid work
		6	Self employed work
pos_paidwrkcor	26. How many hours do they work in a typical week?	User entered integer	
pos_monthcom	27. How much money do they make in a month	User entered integer	
pos_timeoffcom	28. If they took time off from paid work (or business activity if self employed) to come to the facility today approximately how much time did they take off work (or business activity if self-employed)?	User entered integer	
pos_earnlostcor	28a. Did they lose earnings as a result?	1	Yes
		2	No
pos_losthwcom	28b. How much did they lose?	User entered integer	
pos_paybhalfco	28c. Did they have to pay someone to do work on their behalf?	1	Yes
		2	No

Evaluation of coverage and equity of same-day test and treat services (STAT) for the elimination of mother to child transmission of syphilis in Malawi.

Topic guide for qualitative interviews with health care professionals

LANGUAGE: Tumbuka, Chichewa or English (English Version)

AIM: To scope HCW views of potential barriers and facilitators that could influence successful implementation of same day testing and treating for PMTCT of syphilis and routine surveillance of congenital syphilis in Malawi.

STAKEHOLDERS: HCW sampled from ANC and STI settings.

Part 1: Knowledge & Overall Experience

(1) The Malawi National ANC guidelines recommend the following components of care for PMTCT of syphilis

a. Testing at ANC1 –

b. 3 doses of IM benzathine penicillin for positive women. Where possible first dose to be given on the day of diagnosis.

c. Partner notification and testing

d. At each ANC subsequent the testing status of each woman is checked and if it has been missed at ANC 1 it is done that day.

e. Retesting at delivery for any woman who hasn't been tested at ANC1

f. Assessment and management of congenital syphilis at delivery.

What is your experience of delivering each of the components outlined above? (can go through independently). Probe if and how each aspect is done at that centre and any issues that arise regularly for example is treatment co-located with ANC or in a different location? How often is treatment given on the same day as diagnosis?

(2) Describe to me any components of the guideline that you were not aware of before this discussion?

(3) Describe to me the training about MTCT of syphilis that you received? How many health providers have received this training at your facility? What about refresher trainings and how often?

(4) Overall, do you think that PMTCT of syphilis is a priority at your health centre? Why?

Part 2: Barriers / Facilitators Testing & Treatment

(5) In terms of testing and treatment of pregnant women what do you think is working less well at your health centre? (probe ANC/STI location and involvement, logistics, supply and attitudes)

(6) What is your view on willingness of women towards testing and treatment for syphilis during pregnancy?

(7) What kind of partner notification strategies have you experience of? Which ones are in place at this clinic? How do you think partner notification strategies could be improved?

(8) Where do you currently do the testing and treatment? It is recommended that treatment should be given in ANC1? Do you think that would work here? Are there any barriers to the delivery of the treatment being incorporated into routine ANC1 care? Why?

(9) In your opinion, what are the risks to moving treatment of gestational syphilis away from the STI clinic to the ANC or HIV clinic? Why

(10) If women refuse testing and treatment, what kind of reasons do they give?

a. What *cultural beliefs* in Malawi could stand in the way of women accepting syphilis testing or treatment during pregnancy?

b. What *family influences* could potentially stand in the way of women accepting syphilis testing or treatment during pregnancy?

c. What *other factors* could potentially stand in the way of women accepting syphilis testing or treatment during pregnancy?

d. What *cultural beliefs* in Malawi could stand in the way of partner's of pregnant women accepting syphilis testing and treatment?

(11) What factors support the staff in your clinic to deliver PMTCT of syphilis? (them individually, their colleagues)

(12) What factors are hindering in your clinic to deliver PMTCT of syphilis? (them individually, their colleagues)

(13) What support from the government would help sustain PMTCT of syphilis in Malawi?

(14) Are you aware of any initiatives occurring locally to improve services for PMTCT of syphilis?

(15) Do you have a clear system for documenting the treatment that women receive during their pregnancy? Can you describe it? How frequently do you encounter missing or lost documentation among women attending ANC? (probe not having their health passport at delivery, probe mismatch between mother's account of treatment and what is documented.

(16) What is your understanding of the implications of women not receiving adequate treatment during pregnancy? Probe – relevance to the diagnosis of congenital syphilis with respect to date of last dose and number of doses.

(17) What is your understanding of congenital syphilis in newborn babies? What do you know about the treatment they receive? (Do you have a system for identifying babies with congenital syphilis at birth (explore the scenario of asymptomatic babies) Can you describe this system? What treatment do you have available for babies on site? When would you refer a baby for treatment of congenital syphilis?

(18) From your point of view, what can maternity care staff do to encourage more women to attend for treatment of gestational syphilis? What do you think are the major barriers for them attending?

<u>Part 3: Partner notification</u>

(19) What is your understanding of the importance of partner notification?

(20) What is your view on the willingness of partners of positive women towards testing and treatment of syphilis in pregnancy?

(21) What methods do you use to explain the need for partner testing and treatment? What advice do you given women about informing their partners?

(22) Describe to me any experience you may have of women expressing concern about notifying their partners?

(23) Are you aware of any strategies to facilitate the notification of partners locally or anywhere in Malawi i.e. written information?

<u>Part 4: Community perceptions toward syphilis in pregnancy</u>

(24) What perceptions do the local people have of people with syphilis or other STIs?

(25) Can you explain any differences in acceptance of testing for syphilis in pregnancy between women from different socioeconomic groups? (wealthy, professional, skilled, semi-skilled, unemployed, partner, no partner)?

(26) Can you explain any differences in acceptance of testing for syphilis in pregnancy between women of different ages?

(27) Is there anything you would like to add to today's discussion?

(28) Do you have any further questions?

Evaluation of coverage and equity of same-day test and treat services (STAT) for the elimination of mother to child transmission of syphilis in Malawi.

<u>Topic guide for qualitative interviews with pregnant women</u>

LANGUAGE: Tumbuka, Chichewa or English (English Version)

AIM: To scope potential barriers and facilitators experienced by pregnant women with a diagnosis of gestational syphilis which could influence successful completion of their treatment during pregnancy.

STAKEHOLDERS: Pregnant women who have tested positive for syphilis.

<u>Part 1: Demographic data to be collected by interviewer – Fill one for each pregnant woman interviewed.</u>

<u>PID:</u>

(1) Interviewee age:

(2) Marital status:

(3) Experience of stillbirth: YES/NO

(4) Experience of preterm birth: YES/NO

(5) Number of children:

(6) Level of education:

   a. No formal education
   b. Some primary education
   c. Primary Education completed
   d. Secondary education completed
   e. University / College completed
   f. Graduate education /Terminal degree completed.
   g. Other

(7) Does this woman earn any independent income? (i.e. separate to that earned by her partner or parents depending on who she lives with) (Y/N)

(8) If Yes, what is her occupation:

(9) How long does it take her to travel to the facility:

Part 2: Experiences of antenatal care

(1) Tell me a little bit about your current pregnancy so far?

(2) Have you been pregnant before? How many children do you have?

(3) What are your experiences of the antenatal care you have received in this current pregnancy?

(4) What do women in your community / family feel about the care that is offered at the antenatal clinic?

Part 3: Social and cultural factors shaping use and experiences of antenatal care.

(10) What makes it difficult to access and use antenatal services at your nearest health facility? (e.g. transport, distance from healthcare facilities, family structures, fear of healthcare services etc.)

(11) What kinds of things have made it easier for you to use the antenatal services? (e.g. partner, family support, community health worker, being located close to facilities, positive accounts of other people's experiences)

(12) What are other sources of information about pregnancy and pregnancy care? (family, community health worker, books/internet) **Probe: What information do you get/what do they say**

Part 4: Suggestions for improving access to and experiences of antenatal care.

(13) What is your idea of really good antenatal care? If you could change things about the care you have received, what would it be?

(14) What do you think would improve your experiences of the antenatal services?

Part 5: Syphilis diagnosis

(15) What are your thoughts regarding this diagnosis? (probe- ask for the reason for each response mentioned)

(16) How did you feel when you received this diagnosis? Why?

(17) What information was given to you about the disease before you received testing? (probe whether found information useful)

(18) What information was given to you about the disease after you received testing? What additional information would be useful?

(19) What is your understanding of the meaning or implications of syphilis diagnosis (i) for you (ii) for your baby and (iii) for your partner.

(20) What is your understanding of the treatment which will be required now for you? What is you understanding of the treatment that will be required for your baby?

(21) Do you have any concerns about the recommended treatments for you? Do you have any concerns about the recommended treatments for baby?

(22) What are your views regarding the three scheduled treatments? How did you come to the clinic today? Did you have any challenges? Do you think you will be able to access all three treatments? If not, probe why not.

(23) Are you happy to have your syphilis diagnosis and treatments documented in your health passport?

(24) What have you been told today about plans for diagnosis and treatment of your partner?

(25) Have you discussed this diagnosis with your partner yet?

If no: How do you feel about discussing this syphilis diagnosis with your partner? What will you tell him? What do you anticipate your partners reaction will be? Do you anticipate any issues with him attending for treatments?

If yes: How did he react? Did you have any concerns or difficulties in discussing this diagnosis with your partner? Was he able to access treatment? How many times? Where did he access treatment? Did he encounter any issues accessing

(26) Have you had any treatments yet? Were you able to access the recommended treatment? If not, why not? Any difficulties in accessing them? How did you find the treatment? Any side effects?

(27) Were each of your treatments documented in your health passport? Did you mind having this information available in your health passport? Have you changed health passport at any stage in your pregnancy? If yes, why?

(28) Did you discuss your diagnosis with your partner? How did he react? Did you have any concerns or difficulties in discussing this diagnosis with your partner? Was he able to access treatment? How many times? Where did he access treatment? Did he encounter any issues accessing treatment?

(29) Who is involved in decision-making process about your clinic attendance? (Probe: at home or in your village)

(30) If you were to share this information about attending the clinic with anyone at home or in your village, who would that be? Why? What would you tell them?

(31) Do you have any concerns about sharing this diagnosis with anyone at home or in your community? Why? Are you aware of any perceptions in your community associated with a diagnosis of syphilis.

(32) Will you discuss this diagnosis with anyone else? Why?

(33) What suggestions do you have on how to improve the delivery of testing and treatment of syphilis in pregnancy?

(34) Is there anything we have not covered you would like to add?

(35) Do you have any further questions?
