# Supplementary material for: The Road to Elimination of Mother-to-Child Transmission of Syphilis in Malawi: A Mixed-Methods Analysis of Health System Readiness
Source: Sex Transm Dis. 2026 May 18;53(8):492–9. doi: 10.1097/OLQ.0000000000002359 (PMC13326934; doi:10.1097/OLQ.0000000000002359)
Supplement: Supplementary file 3 [file std-53-492-s003.pdf]

```r


## ----setup-----------------------------------------------------------------------------------------------------------------------
#setwd("../Data/07_Mar_2023_06_35_41_syph_stat_dataset")

rm(list = ls())


library("tidyverse")
library("haven")
library("readxl")
library("sjlabelled") #for factor labels
library("anytime") #for the other types of dates
library("kableExtra")
library("sf")
library("janitor")
library("FactoMineR")
library("factoextra")
library("table1")
library("epitools")


##Objective 1


## ----loaddata--------------------------------------------------------------------------------------------------------------------
Bln_mlw <- read_dta("SyphStat_Baseline.dta")

#I have added new variables for HC administration (bas_admin) and location (bas_location) in this updated baseline dataset

Bln_mlw_up <- read.csv("SyphStat_Baseline_label_updated.csv")



## ----Tables----------------------------------------------------------------------------------------------------------------------

##Facility demographics

#Health centre region
table(Bln_mlw_up$bas_region)

#ANC sessions
Bln_mlw <- Bln_mlw %>%
  dplyr::mutate(
    bas_mon = ifelse(bas_monanac %in% c(1, 2), 1, ifelse(bas_monanac == 3, 2, 0)),
    bas_tue = ifelse(bas_tueanc %in% c(1, 2), 1, ifelse(bas_tueanc == 3, 2, 0)),
    bas_wed = ifelse(bas_wedanc %in% c(1, 2), 1, ifelse(bas_wedanc == 3, 2, 0)),
    bas_thu = ifelse(bas_thuranc %in% c(1, 2), 1, ifelse(bas_thuranc == 3, 2, 0)),
    bas_fri = ifelse(bas_frianc %in% c(1, 2), 1, ifelse(bas_frianc == 3, 2, 0))
  ) %>%
  dplyr::mutate(
    anc_sessions = rowSums(select(Bln_mlw, bas_mon:bas_fri))
  )
table(Bln_mlw$anc_sessions)


##ANC services

#Method of syphilis testing offered
```

```
table(Bln_mlw$bas_vdrl)

table(Bln_mlw$bas_rpr)

table(Bln_mlw$bas_rpoct)

table(Bln_mlw$bas_prick)

table(Bln_mlw$bas_vdrlrpr)

table(Bln_mlw$bas_rprpoct)

table(Bln_mlw$bas_vdrlother)

#Location of syphilis testing

table(Bln_mlw$bas_syphtest)

table(Bln_mlw$bas_syphpreg)

#Where is IM BPG administered

table(Bln_mlw$bas_rxwhere)


#If treatment not available where is treatment advised
table(Bln_mlw$bas_rxyes)

#Offer partner notification
table(Bln_mlw$bas_partner)

#Where are partners treated
table(Bln_mlw$bas_rxpart)




##Identification of CS at birth

#Routine re-testing of women at delivery
table(Bln_mlw$bas_syphdel)

#Untested women tested at delivery
table(Bln_mlw$bas_syphretest)

#Syphilis treatment status review at delivery
table(Bln_mlw$bas_rxteview)

#Which infants receive treatment
table(Bln_mlw$bas_congesyph)

table(Bln_mlw$bas_congespec)

#What treatments are offered to infants
table(Bln_mlw$bas_statdose)

table(Bln_mlw$bas_tendoses)

table(Bln_mlw$bas_hosrefer)

table(Bln_mlw$bas_seekcare)

table(Bln_mlw$bas_otherneo)

table(Bln_mlw$bas_neomgtspec)
```

```
##Surveillance and data capture

#How is clinical data captured at ANC
table(Bln_mlw$bas_emr)

table(Bln_mlw$bas_hmis)

table(Bln_mlw$bas_hpassport)

table(Bln_mlw$bas_hcw)

table(Bln_mlw$bas_combiemr)

table(Bln_mlw$bas_dataother)

table(Bln_mlw$bas_dataspec)

#Where is maternal syphilis treatment captured
table(Bln_mlw$bas_ancdata)

table(Bln_mlw$bas_ancpspt)

table(Bln_mlw$bas_stiregdoc)

table(Bln_mlw$bas_otherrec)

table(Bln_mlw$bas_recspec)

#If women buy the drug, where is it captured
table(Bln_mlw$bas_stireg)

table(Bln_mlw$bas_stiemr)

table(Bln_mlw$bas_stipspt)

table(Bln_mlw$bas_stiother)

table(Bln_mlw$bas_stiospec)

#How is treatment captured in HMIS
table(Bln_mlw$bas_hmisreport)

#If an infant is diagnosed with CS where is it recorded
table(Bln_mlw$bas_mreg)

table(Bln_mlw$bas_pnreg)

table(Bln_mlw$bas_mpspt)

table(Bln_mlw$bas_neopspt)

table(Bln_mlw$bas_otherreg)

table(Bln_mlw$bas_regspec)

table(Bln_mlw$bas_comment)



#-----------------------------------------------------------------------------------------------------------------------------

## Objective 2

```

```r
## ----loaddata---------------------------------------------------------------------------------------------------------------
#Load MLW data
ANC_mlw <- read_dta("SyphStat_AntenatalANC1.dta")
ANCsub_mlw <- read_dta("SyphStat_SubsequentANC.dta")
DEL_mlw <- read_dta("SyphStat_Delivery.dta")

#Load Meiru data
ANC_meiru <- read_dta("Syph_Antenatal_Meiru.dta")
Ante_meiru <- read_dta("MEIRU_ANC.dta")
DEL_meiru <- read_dta("Syph_Delivery_Meiru.dta")


## ----processdata------------------------------------------------------------------------------------------------------------
#Meiru data

#ANC_meiru_distinct <- ANC_meiru[!duplicated(ANC_meiru$pid),]
ANC_meiru$data_date <- as.Date(ANC_meiru$data_date)

Ante_meiru_sub <- Ante_meiru %>% dplyr:: select(data_date, pid, f_id, hiv, seros)
#Ante_meiru_sub <- Ante_meiru_sub[!duplicated(Ante_meiru_sub$pid),]
Ante_meiru_sub <- Ante_meiru_sub %>% mutate(
  data_date=ifelse(grepl("-", data_date), format(anytime(data_date), "%Y-%m-%d"), format(dmy(data_date)))
)
Ante_meiru_sub$data_date <- as.Date(Ante_meiru_sub$data_date)

ANC_meiru_final <- left_join(ANC_meiru, Ante_meiru_sub, by=c("data_date","pid", "f_id"))
ANC_meiru_final$f_id <- as.factor(ANC_meiru_final$f_id)
ANC_meiru_final$f_id <- set_labels(ANC_meiru_final$f_id, labels = c("Mpemba heath centre", "South Lunzu health centre", "Zingwangwa health centre", "Area 25 health centre",
                                                                    "Chilumba community hospital", "Karonga District Hospital"))
ANC_meiru_final$data_date <- as.Date(ANC_meiru_final$data_date)

ANC_meiru_final <- ANC_meiru_final %>%
  select(-crf_ver, -start, -end, -deviceid, -pin, -project_dsid)


## ----prop28-----------------------------------------------------------------------------------------------------------------

#Total
ANCsub_mlw <- ANCsub_mlw %>%
  mutate(
    anc_anc1dt = lubridate::dmy(anc_anc1dt),
    diffDate_anc1 = difftime(data_date, anc_anc1dt, units = "weeks"),
    diffwks_anc1 = anc_gestationw-diffDate_anc1,
    prop_28 = ifelse(((anc_ancvisit==1 | (is.na(anc_ancvisit))) & anc_gestationw < 28) | (anc_ancvisit>1 & diffwks_anc1 < 28), 1, 0)
  )


ANC_mlw <- ANC_mlw %>%
  mutate(
    prop_28 = ifelse(gest_age_w < 28, 1, 0),
    prop_28_N = ifelse(f_id==203 & gest_age_w < 28, 1,0),
    prop_28_S = ifelse(f_id %in% 101:103 & gest_age_w < 28, 1, 0)
  )

```

```
ANC_meiru_final <- ANC_meiru_final %>%
  mutate(
    prop_28 = ifelse(gest_age_w < 28, 1, 0),
    prop_28_N = ifelse(f_id %in% c(202,203) & gest_age_w < 28, 1,0),
    prop_28_C = ifelse(f_id==201 & gest_age_w < 28, 1, 0),
    prop_28_S = ifelse(f_id %in% 101:103 & gest_age_w < 28, 1, 0)
  )


MLWprop_28 <- ((sum(na.omit(ANCsub_mlw$prop_28)) + sum(na.omit(ANC_mlw$prop_28)) + sum(na.omit(ANC_meiru_final$prop_28)))/
                 (nrow(ANCsub_mlw) + nrow(ANC_mlw) + nrow(ANC_meiru_final))) * 100

CI_MLWprop_28 <- prop.test(x=sum(na.omit(ANCsub_mlw$prop_28)) + sum(na.omit(ANC_mlw$prop_28)) + sum(na.omit(ANC_meiru_final$prop_28)), n=nrow(ANCsub_mlw) + nrow(ANC_mlw) + nrow(ANC_meiru_final),
                           conf.level = .95, correct=FALSE)

#By region
#North
MLWprop_28_N <- ((sum(na.omit(ANC_mlw$prop_28_N)) + sum(na.omit(ANC_meiru_final$prop_28_N)))/
                   (nrow(filter(ANC_mlw, f_id==203)) + nrow(filter(ANC_meiru_final, f_id %in% c(202,203))))) * 100

CI_MLWprop_28_N <- prop.test(x=sum(na.omit(ANC_mlw$prop_28_N)) + sum(na.omit(ANC_meiru_final$prop_28_N)), n=nrow(filter(ANC_mlw, f_id==203)) + nrow(filter(ANC_meiru_final, f_id %in% c(202,203))),
                             conf.level = .95, correct=FALSE)

#Central
MLWprop_28_C <- (sum(na.omit(ANC_meiru_final$prop_28_C))/ nrow(filter(ANC_meiru_final, f_id==201))) * 100

CI_MLWprop_28_C <- prop.test(x=sum(na.omit(ANC_meiru_final$prop_28_C)), n=nrow(filter(ANC_meiru_final, f_id==201)),
                             conf.level = .95, correct=FALSE)

#Southern
MLWprop_28_S <- ((sum(na.omit(ANCsub_mlw$prop_28))+sum(na.omit(ANC_mlw$prop_28_S)) + sum(na.omit(ANC_meiru_final$prop_28_S)))/
                   (nrow(ANCsub_mlw) + nrow(filter(ANC_mlw, f_id %in% 101:103)) + nrow(filter(ANC_meiru_final, f_id %in% 101:103)))) * 100

CI_MLWprop_28_S <- prop.test(x=sum(na.omit(ANCsub_mlw$prop_28))+sum(na.omit(ANC_mlw$prop_28_S)) + sum(na.omit(ANC_meiru_final$prop_28_S)), n=nrow(ANCsub_mlw) + nrow(filter(ANC_mlw, f_id %in% 101:103)) + nrow(filter(ANC_meiru_final, f_id %in% 101:103)),
                             conf.level = .95, correct=FALSE)



## ----propsyph28----------------------------------------------------------------------------------------------------------------
ANCsub_mlw  <- ANCsub_mlw %>%
  mutate(
    prop_syph28 = ifelse(prop_28==1 & anc_syphdoc==1, 1, 0)
  )

ANC_mlw$prop_syph28 <- ifelse(ANC_mlw$prop_28==1 & ANC_mlw$syphillis==1, 1, 0)

ANC_meiru_final$prop_syph28 <- ifelse(ANC_meiru_final$prop_28 & ANC_meiru_final$syphillis==1, 1, 0)


MLWprop_syph28 <- ((sum(na.omit(ANCsub_mlw$prop_syph28==1)) + sum(na.omit(ANC_mlw$
```

```r
prop_syph28==1)) + sum(na.omit(ANC_meiru_final$prop_syph28))) /
                        (nrow(ANCsub_mlw) + nrow(ANC_mlw) + nrow(ANC_meiru_final))) * 100

CI_MLWprop_syph28 <- prop.test(x=sum(na.omit(ANCsub_mlw$prop_syph28==1)) + sum(na.omit(ANC_mlw$prop_syph28==1)) + sum(na.omit(ANC_meiru_final$prop_syph28)), n=nrow(ANCsub_mlw) + nrow(ANC_mlw) + nrow(ANC_meiru_final),
                               conf.level = .95, correct=FALSE)

#By Region
#North
MLWprop_syph28_N <- ((sum(na.omit(filter(ANC_mlw, f_id==203)$prop_syph28)) + sum(na.omit(filter(ANC_meiru_final, f_id %in% c(202,203))$prop_syph28)))/
                       (nrow(filter(ANC_mlw, f_id==203)) + nrow(filter(ANC_meiru_final, f_id %in% c(202,203))))) * 100

CI_MLWprop_syph28_N <- prop.test(x=sum(na.omit(filter(ANC_mlw, f_id==203)$prop_syph28)) + sum(na.omit(filter(ANC_meiru_final, f_id %in% c(202,203))$prop_syph28)), n=nrow(filter(ANC_mlw, f_id==203)) + nrow(filter(ANC_meiru_final, f_id %in% c(202,203))),
                                 conf.level = .95, correct=FALSE)


#Central
MLWprop_syph28_C <- (sum(na.omit(filter(ANC_meiru_final, f_id==201)$prop_syph28))/
                       nrow(filter(ANC_meiru_final, f_id==201))) * 100

CI_MLWprop_syph28_C <- prop.test(x=sum(na.omit(filter(ANC_meiru_final, f_id==201)$prop_syph28)), n=nrow(filter(ANC_meiru_final, f_id==201)),
                                 conf.level = .95, correct=FALSE)

#South
MLWprop_syph28_S <- ((sum(na.omit(ANCsub_mlw$prop_syph28))+sum(na.omit(filter(ANC_mlw, f_id %in% 101:103)$prop_syph28)) + sum(na.omit(filter(ANC_meiru_final, f_id %in% 101:103)$prop_syph28)))/
                       (nrow(ANCsub_mlw) + nrow(filter(ANC_mlw, f_id %in% 101:103)) +
                       nrow(filter(ANC_meiru_final, f_id %in% 101:103)))) * 100

CI_MLWprop_syph28_S <- prop.test(x=sum(na.omit(ANCsub_mlw$prop_syph28))+sum(na.omit(filter(ANC_mlw, f_id %in% 101:103)$prop_syph28)) + sum(na.omit(filter(ANC_meiru_final, f_id %in% 101:103)$prop_syph28)), n=nrow(ANCsub_mlw) + nrow(filter(ANC_mlw, f_id %in% 101:103)) + nrow(filter(ANC_meiru_final, f_id %in% 101:103)),
                                 conf.level = .95, correct=FALSE)



## ----propsyph-------------------------------------------------------------------------------------------------------------------
ANCsub_mlw$prop_syph <- ifelse(ANCsub_mlw$anc_syphdoc==1, 1, 0)

ANC_mlw$prop_syph <- ifelse(ANC_mlw$syphillis==1, 1, 0)

ANC_meiru_final$prop_syph <- ifelse(ANC_meiru_final$syphillis==1, 1, 0)


MLWprop_syph <- ((sum(na.omit(ANCsub_mlw$prop_syph)) + sum(na.omit(ANC_mlw$prop_syph)) + sum(na.omit(ANC_meiru_final$prop_syph))) /
                   (nrow(ANCsub_mlw) + nrow(ANC_mlw) + nrow(ANC_meiru_final))) * 100

CI_MLWprop_syph <- prop.test(x=sum(na.omit(ANCsub_mlw$prop_syph==1)) + sum(na.omit(ANC_mlw$prop_syph==1)) + sum(na.omit(ANC_meiru_final$prop_syph)), n=nrow(ANCsub_mlw) + nrow(ANC_mlw) + nrow(ANC_meiru_final),
                             conf.level = .95, correct=FALSE)

#By Region
#North
MLWprop_syph_N <- ((sum(na.omit(filter(ANC_mlw, f_id==203)$prop_syph)) + sum(na.omit(
```

```
filter(ANC_meiru_final, f_id %in% c(202,203))$prop_syph)))/
                     (nrow(filter(ANC_mlw, f_id==203)) + nrow(filter(ANC_meiru_final,
                     f_id %in% c(202,203))))) * 100

CI_MLWprop_syph_N <- prop.test(x=sum(na.omit(filter(ANC_mlw, f_id==203)$prop_syph)) + sum
(na.omit(filter(ANC_meiru_final, f_id %in% c(202,203))$prop_syph)), n=nrow(filter(ANC_mlw
, f_id==203)) + nrow(filter(ANC_meiru_final, f_id %in% c(202,203))),
                               conf.level = .95, correct=FALSE)



#Central
MLWprop_syph_C <- (sum(na.omit(filter(ANC_meiru_final, f_id==201)$prop_syph))/
                     nrow(filter(ANC_meiru_final, f_id==201))) * 100

CI_MLWprop_syph_C <- prop.test(x=sum(na.omit(filter(ANC_meiru_final, f_id==201)$prop_syph
)), n=nrow(filter(ANC_meiru_final, f_id==201)),
                               conf.level = .95, correct=FALSE)

#South
MLWprop_syph_S <- ((sum(na.omit(ANCsub_mlw$prop_syph))+sum(na.omit(filter(ANC_mlw, f_id
%in% 101:103)$prop_syph)) + sum(na.omit(filter(ANC_meiru_final, f_id %in% 101:103)$
prop_syph)))/
                     (nrow(ANCsub_mlw) + nrow(filter(ANC_mlw, f_id %in% 101:103)) + nrow(
                     filter(ANC_meiru_final, f_id %in% 101:103)))) * 100

CI_MLWprop_syph_S <- prop.test(x=sum(na.omit(ANCsub_mlw$prop_syph))+sum(na.omit(filter(
ANC_mlw, f_id %in% 101:103)$prop_syph)) + sum(na.omit(filter(ANC_meiru_final, f_id %in%
101:103)$prop_syph)), n=nrow(ANCsub_mlw) + nrow(filter(ANC_mlw, f_id %in% 101:103)) +
nrow(filter(ANC_meiru_final, f_id %in% 101:103)),
                               conf.level = .95, correct=FALSE)




##
----prophiv28-------------------------------------------------------------------------
-----------------------------------
ANCsub_mlw  <- ANCsub_mlw %>%
  mutate(
    prop_hiv28 = ifelse(prop_28==1 & anc_hprec==1, 1, 0)
  )

ANC_mlw$prop_hiv28 <- ifelse(ANC_mlw$prop_28==1 & ANC_mlw$hiv==1, 1, 0)

ANC_meiru_final$prop_hiv28 <- ifelse(ANC_meiru_final$prop_28 & ANC_meiru_final$hiv==1, 1
, 0)


MLWprop_hiv28 <- ((sum(na.omit(ANCsub_mlw$prop_hiv28==1)) + sum(na.omit(ANC_mlw$
prop_hiv28==1)) + sum(na.omit(ANC_meiru_final$prop_hiv28))) /
                   (nrow(ANCsub_mlw) + nrow(ANC_mlw) + nrow(ANC_meiru_final))) * 100

CI_MLWprop_hiv28 <- prop.test(x=sum(na.omit(ANCsub_mlw$prop_hiv28==1)) + sum(na.omit(
ANC_mlw$prop_hiv28==1)) + sum(na.omit(ANC_meiru_final$prop_hiv28)), n=nrow(ANCsub_mlw) +
nrow(ANC_mlw) + nrow(ANC_meiru_final),
                              conf.level = .95, correct=FALSE)

#By Region
#North
MLWprop_hiv28_N <- ((sum(na.omit(filter(ANC_mlw, f_id==203)$prop_hiv28)) + sum(na.omit(
filter(ANC_meiru_final, f_id %in% c(202,203))$prop_hiv28)))/
                      (nrow(filter(ANC_mlw, f_id==203)) + nrow(filter(ANC_meiru_final,
                      f_id %in% c(202,203))))) * 100

CI_MLWprop_hiv28_N <- prop.test(x=sum(na.omit(filter(ANC_mlw, f_id==203)$prop_hiv28)) +
sum(na.omit(filter(ANC_meiru_final, f_id %in% c(202,203))$prop_hiv28)), n=nrow(filter(
```

```
ANC_mlw, f_id==203)) + nrow(filter(ANC_meiru_final, f_id %in% c(202,203))),
                                conf.level = .95, correct=FALSE)


#Central
MLWprop_hiv28_C <- (sum(na.omit(filter(ANC_meiru_final, f_id==201)$prop_hiv28))/
                      nrow(filter(ANC_meiru_final, f_id==201))) * 100

CI_MLWprop_hiv28_C <- prop.test(x=sum(na.omit(filter(ANC_meiru_final, f_id==201)$
prop_hiv28)), n=nrow(filter(ANC_meiru_final, f_id==201)),
                                conf.level = .95, correct=FALSE)

#South
MLWprop_hiv28_S <- ((sum(na.omit(ANCsub_mlw$prop_hiv28))+sum(na.omit(filter(ANC_mlw, f_id
 %in% 101:103)$prop_hiv28)) + sum(na.omit(filter(ANC_meiru_final, f_id %in% 101:103)$
prop_hiv28)))/
                      (nrow(ANCsub_mlw) + nrow(filter(ANC_mlw, f_id %in% 101:103)) + nrow
                      (filter(ANC_meiru_final, f_id %in% 101:103)))) * 100

CI_MLWprop_hiv28_S <- prop.test(x=sum(na.omit(ANCsub_mlw$prop_hiv28))+sum(na.omit(filter(
ANC_mlw, f_id %in% 101:103)$prop_hiv28)) + sum(na.omit(filter(ANC_meiru_final, f_id %in%
101:103)$prop_hiv28)), n=nrow(ANCsub_mlw) + nrow(filter(ANC_mlw, f_id %in% 101:103)) +
nrow(filter(ANC_meiru_final, f_id %in% 101:103)),
                                conf.level = .95, correct=FALSE)



##
----prophiv---------------------------------------------------------------------------
-----------------------------------

ANCsub_mlw$prop_hiv <- ifelse(ANCsub_mlw$anc_hprec==1, 1, 0)

ANC_mlw$prop_hiv <- ifelse(ANC_mlw$hiv==1, 1, 0)

ANC_meiru_final$prop_hiv <- ifelse(ANC_meiru_final$hiv==1, 1, 0)


MLWprop_hiv <- ((sum(na.omit(ANCsub_mlw$prop_hiv)) + sum(na.omit(ANC_mlw$prop_hiv)) + sum
(na.omit(ANC_meiru_final$prop_hiv))) /
                  (nrow(ANCsub_mlw) + nrow(ANC_mlw) + nrow(ANC_meiru_final))) * 100

CI_MLWprop_hiv <- prop.test(x=sum(na.omit(ANCsub_mlw$prop_hiv)) + sum(na.omit(ANC_mlw$
prop_hiv)) + sum(na.omit(ANC_meiru_final$prop_hiv)), n=nrow(ANCsub_mlw) + nrow(ANC_mlw) +
 nrow(ANC_meiru_final),
                            conf.level = .95, correct=FALSE)


#By Region
#North
MLWprop_hiv_N <- ((sum(na.omit(filter(ANC_mlw, f_id==203)$prop_hiv)) + sum(na.omit(filter
(ANC_meiru_final, f_id %in% c(202,203))$prop_hiv)))/
                    (nrow(filter(ANC_mlw, f_id==203)) + nrow(filter(ANC_meiru_final, f_id
                     %in% c(202,203))))) * 100

CI_MLWprop_hiv_N <- prop.test(x=sum(na.omit(filter(ANC_mlw, f_id==203)$prop_hiv)) + sum(
na.omit(filter(ANC_meiru_final, f_id %in% c(202,203))$prop_hiv)), n=nrow(filter(ANC_mlw,
f_id==203)) + nrow(filter(ANC_meiru_final, f_id %in% c(202,203))),
                              conf.level = .95, correct=FALSE)


#Central
MLWprop_hiv_C <- (sum(na.omit(filter(ANC_meiru_final, f_id==201)$prop_hiv))/
                    nrow(filter(ANC_meiru_final, f_id==201))) * 100

CI_MLWprop_hiv_C <- prop.test(x=sum(na.omit(filter(ANC_meiru_final, f_id==201)$prop_hiv))
```

```
, n=nrow(filter(ANC_meiru_final, f_id==201)),
                              conf.level = .95, correct=FALSE)

#South
MLWprop_hiv_S <- ((sum(na.omit(ANCsub_mlw$prop_hiv))+sum(na.omit(filter(ANC_mlw, f_id
%in% 101:103)$prop_hiv)) + sum(na.omit(filter(ANC_meiru_final, f_id %in% 101:103)$
prop_hiv)))/
                    (nrow(ANCsub_mlw) + nrow(filter(ANC_mlw, f_id %in% 101:103)) + nrow(
                    filter(ANC_meiru_final, f_id %in% 101:103)))) * 100

CI_MLWprop_hiv_S <- prop.test(x=sum(na.omit(ANCsub_mlw$prop_hiv))+sum(na.omit(filter(
ANC_mlw, f_id %in% 101:103)$prop_hiv)) + sum(na.omit(filter(ANC_meiru_final, f_id %in%
101:103)$prop_hiv)), n=nrow(ANCsub_mlw) + nrow(filter(ANC_mlw, f_id %in% 101:103)) + nrow
(filter(ANC_meiru_final, f_id %in% 101:103)),
                              conf.level = .95, correct=FALSE)



##
----propsyphtrt------------------------------------------------------------------------
------------------------------------

##### create time difference variables

DEL_mlw<-DEL_mlw %>%
  dplyr::mutate(
    data_date=as.Date(data_date),
    del_inj1dt=lubridate::dmy(del_inj1dt),
    del_inj2dt=lubridate::dmy(del_inj2dt),
    del_inj3dt=lubridate::dmy(del_inj3dt),
  ) %>%
  dplyr::mutate(
    diffDate_inj1dt=data_date-del_inj1dt,
    diffDate_inj2dt=data_date-del_inj2dt,
    diffDate_inj3dt=data_date-del_inj3dt
  )


DEL_mlw$mindt <- apply(DEL_mlw[, c("del_inj1dt", "del_inj2dt", "del_inj3dt")], 1,
function(x) min(x, na.rm = TRUE))
DEL_mlw$mindt <- as.Date(DEL_mlw$mindt)
DEL_mlw$diffDate <- DEL_mlw$data_date - DEL_mlw$mindt


DEL_mlw <- DEL_mlw %>% mutate(
  prop_syphtrt=ifelse(del_result==1 &  del_treatment==1 & (del_injections==1 |
  del_injections==2 | del_injections==3) &
                       (del_inj1doc==1 | del_inj2doc==1 | del_inj3doc==1) & diffDate>=30
                       , 1, 0),

  prop_syphtrt3=ifelse(del_result==1 & del_treatment==1 & del_injections==3 & del_inj3doc
  ==1 & diffDate_inj3dt>=30, 1, 0)
)




####################################

DEL_meiru<-DEL_meiru %>%
  dplyr::mutate(
    data_date=as.Date(data_date),
    del_dt1=lubridate::dmy(del_dt1),
    del_dt2=lubridate::dmy(del_dt2),
    del_dt3=lubridate::dmy(del_dt3),
  ) %>%
```

```r
  dplyr::mutate(
    diffDate_dt1=data_date-del_dt1,
    diffDate_dt2=data_date-del_dt2,
    diffDate_dt3=data_date-del_dt3
  )


DEL_meiru$mindt <- apply(DEL_meiru[, c("del_dt1", "del_dt2", "del_dt3")], 1, function(x) min(x, na.rm = TRUE))
DEL_meiru$mindt <- ymd(DEL_meiru$mindt)
DEL_meiru$diffDate <- DEL_meiru$data_date - DEL_meiru$mindt


DEL_meiru <- DEL_meiru %>% mutate(
  prop_syphtrt=ifelse(del_result==1 &  del_rx==1 & (del_treat==1 | del_treat==2 | del_treat==3) &
                      (del_dt1doc==1 | del_dt2doc==1 | del_dt3doc==1) & diffDate>=30, 1, 0),

  prop_syphtrt3=ifelse(del_result==1 & del_rx==1 & del_treat==3 & del_dt3doc==1 & diffDate_dt3>=30, 1, 0)
)

######################################################################################

MLWprop_syphtrt <- ((sum(na.omit(DEL_mlw$prop_syphtrt)) + sum(na.omit(DEL_meiru$prop_syphtrt))) / (sum(na.omit(DEL_mlw$del_result)) + sum(na.omit(DEL_meiru$del_result)))) * 100

CI_MLWprop_syphtrt <- prop.test(x=sum(na.omit(DEL_mlw$prop_syphtrt)) + sum(na.omit(DEL_meiru$prop_syphtrt)), n=sum(na.omit(DEL_mlw$del_result)) + sum(na.omit(DEL_meiru$del_result)), conf.level = .95, correct=FALSE)


MLWprop_syphtrt3 <- ((sum(na.omit(DEL_mlw$prop_syphtrt3)) + sum(na.omit(DEL_meiru$prop_syphtrt3))) / (sum(na.omit(DEL_mlw$del_result)) + sum(na.omit(DEL_meiru$del_result)))) * 100

CI_MLWprop_syphtrt3 <- prop.test(x=sum(na.omit(DEL_mlw$prop_syphtrt3)) + sum(na.omit(DEL_meiru$prop_syphtrt3)), n=sum(na.omit(DEL_mlw$del_result)) + sum(na.omit(DEL_meiru$del_result)),
                                 conf.level = .95, correct=FALSE)


#By region
#North
MLWprop_syphtrt_N <- (sum(na.omit(filter(DEL_meiru, f_id %in% c(202,203))$prop_syphtrt)) / sum(na.omit(filter(DEL_meiru, f_id %in% c(202,203))$del_result))) * 100

CI_MLWprop_syphtrt_N <- prop.test(x=sum(na.omit(filter(DEL_meiru, f_id %in% c(202,203))$prop_syphtrt)) , n=sum(na.omit(filter(DEL_meiru, f_id %in% c(202,203))$del_result)), conf.level = .95, correct=FALSE)


MLWprop_syphtrt3_N <- (sum(na.omit(filter(DEL_meiru, f_id %in% c(202,203))$prop_syphtrt3)) / sum(na.omit(filter(DEL_meiru, f_id %in% c(202,203))$del_result))) * 100

CI_MLWprop_syphtrt3_N <- prop.test(x=sum(na.omit(filter(DEL_meiru, f_id %in% c(202,203))$prop_syphtrt3)) , n=sum(na.omit(filter(DEL_meiru, f_id %in% c(202,203))$del_result)), conf.level = .95, correct=FALSE)


#Central
MLWprop_syphtrt_C <- ((sum(na.omit(filter(DEL_mlw, del_fid==201)$prop_syphtrt)) + sum(na.omit(filter(DEL_meiru, f_id==201)$prop_syphtrt))) / (sum(na.omit(filter(DEL_mlw, del_fid==201)$del_result)) + sum(na.omit(filter(DEL_meiru, f_id==201)$del_result)))) *
```

```
100

CI_MLWprop_syphtrt_C <- prop.test(x=sum(na.omit(filter(DEL_mlw, del_fid==201)$prop_syphtrt)) + sum(na.omit(filter(DEL_meiru, f_id==201)$prop_syphtrt)) , n=sum(na.omit(filter(DEL_mlw, del_fid==201)$del_result)) + sum(na.omit(filter(DEL_meiru, f_id==201)$del_result)), conf.level = .95, correct=FALSE)

MLWprop_syphtrt3_C <- ((sum(na.omit(filter(DEL_mlw, del_fid==201)$prop_syphtrt3)) + sum(na.omit(filter(DEL_meiru, f_id==201)$prop_syphtrt3))) / (sum(na.omit(filter(DEL_mlw, del_fid==201)$del_result)) + sum(na.omit(filter(DEL_meiru, f_id==201)$del_result)))) * 100

CI_MLWprop_syphtrt3_C <- prop.test(x=sum(na.omit(filter(DEL_mlw, del_fid==201)$prop_syphtrt3)) + sum(na.omit(filter(DEL_meiru, f_id==201)$prop_syphtrt3)) , n=sum(na.omit(filter(DEL_mlw, del_fid==201)$del_result)) + sum(na.omit(filter(DEL_meiru, f_id==201)$del_result)), conf.level = .95, correct=FALSE)

#South
MLWprop_syphtrt_S <- (sum(na.omit(filter(DEL_mlw, del_fid %in% 101:104)$prop_syphtrt)) / sum(na.omit(filter(DEL_mlw, del_fid %in% 101:104)$del_result))) * 100

CI_MLWprop_syphtrt_S <- prop.test(x=sum(na.omit(filter(DEL_mlw, del_fid %in% 101:104)$prop_syphtrt)) , n=sum(na.omit(filter(DEL_mlw, del_fid %in% 101:104)$del_result)), conf.level = .95, correct=FALSE)

MLWprop_syphtrt3_S <- (sum(na.omit(filter(DEL_mlw, del_fid %in% 101:104)$prop_syphtrt3)) / sum(na.omit(filter(DEL_mlw, del_fid %in% 101:104)$del_result))) * 100

CI_MLWprop_syphtrt3_S <- prop.test(x=sum(na.omit(filter(DEL_mlw, del_fid %in% 101:104)$prop_syphtrt3)) , n=sum(na.omit(filter(DEL_mlw, del_fid %in% 101:104)$del_result)), conf.level = .95, correct=FALSE)



## ----propstable------------------------------------------------------------------------------------------------------------

props_table <- data.frame(Outcomes = c("Attended ANC < 28 weeks", "Tested for syphilis < 28 weeks","Tested for syphilis in pregnancy","Tested for HIV < 28 weeks", "Tested for HIV in pregnancy", "Received 1 dose of BPG", "Received 3 doses of BPG"),
                          Percent = round (c(MLWprop_28, MLWprop_syph28,MLWprop_syph, MLWprop_hiv28, MLWprop_hiv, MLWprop_syphtrt, MLWprop_syphtrt3), 2))



props_table <- props_table %>%
  mutate(
    CI=c(paste(round(CI_MLWprop_28$conf.int[1] * 100,2),round(CI_MLWprop_28$conf.int[2]* 100, 2),sep = ", "),
         paste(round(CI_MLWprop_syph28$conf.int[1]* 100,2),round(CI_MLWprop_syph28$conf.int[2]* 100,2),sep = ", "),
         paste(round(CI_MLWprop_syph$conf.int[1]* 100,2),round(CI_MLWprop_syph$conf.int[2]* 100,2),sep = ", "),
         paste(round(CI_MLWprop_hiv28$conf.int[1]* 100,2),round(CI_MLWprop_hiv28$conf.int[2]* 100,2),sep = ", "),
         paste(round(CI_MLWprop_hiv$conf.int[1]* 100,2),round(CI_MLWprop_hiv$conf.int[2]* 100,2),sep = ", "),
         paste(round(CI_MLWprop_syphtrt$conf.int[1]* 100,2),round(CI_MLWprop_syphtrt$conf.int[2]* 100,2),sep = ", "),
         paste(round(CI_MLWprop_syphtrt3$conf.int[1]* 100,2),round(CI_MLWprop_syphtrt3$conf.int[2]* 100,2),sep = ", ")),

    number = c(sum(na.omit(ANCsub_mlw$prop_28)) + sum(na.omit(ANC_mlw$prop_28)) + sum(na.omit(ANC_meiru_final$prop_28)),
               sum(na.omit(ANCsub_mlw$prop_syph28)) + sum(na.omit(ANC_mlw$prop_syph28)) +
```

```r
                 sum(na.omit(ANC_meiru_final$prop_syph28)),
                 sum(na.omit(ANCsub_mlw$prop_syph)) + sum(na.omit(ANC_mlw$prop_syph)) + sum(na.omit(ANC_meiru_final$prop_syph)),
                 sum(na.omit(ANCsub_mlw$prop_hiv28)) + sum(na.omit(ANC_mlw$prop_hiv28)) + sum(na.omit(ANC_meiru_final$prop_hiv28)),
                 sum(na.omit(ANCsub_mlw$prop_hiv)) + sum(na.omit(ANC_mlw$prop_hiv)) + sum(na.omit(ANC_meiru_final$prop_hiv)),
                 sum(na.omit(DEL_mlw$prop_syphtrt)) + sum(na.omit(DEL_meiru$prop_syphtrt)),
                 sum(na.omit(DEL_mlw$prop_syphtrt3)) + sum(na.omit(DEL_meiru$prop_syphtrt3))),

    total = c(nrow(ANCsub_mlw) + nrow(ANC_mlw) + nrow(ANC_meiru_final),
              nrow(ANCsub_mlw) + nrow(ANC_mlw) + nrow(ANC_meiru_final),
              nrow(ANCsub_mlw) + nrow(ANC_mlw) + nrow(ANC_meiru_final),
              nrow(ANCsub_mlw) + nrow(ANC_mlw) + nrow(ANC_meiru_final),
              nrow(ANCsub_mlw) + nrow(ANC_mlw) + nrow(ANC_meiru_final),
              sum(na.omit(DEL_mlw$del_result)) + sum(na.omit(DEL_meiru$del_result)),
              sum(na.omit(DEL_mlw$del_result)) + sum(na.omit(DEL_meiru$del_result))),

    NAs = c(sum(is.na(ANCsub_mlw$prop_28)) + sum(is.na(ANC_mlw$prop_28)) + sum(is.na(ANC_meiru_final$prop_28)),
            sum(is.na(ANCsub_mlw$prop_syph28)) + sum(is.na(ANC_mlw$prop_syph28)) + sum(is.na(ANC_meiru_final$prop_syph28)),
            sum(is.na(ANCsub_mlw$prop_syph)) + sum(is.na(ANC_mlw$prop_syph)) + sum(is.na(ANC_meiru_final$prop_syph)),
            sum(is.na(ANCsub_mlw$prop_hiv28)) + sum(is.na(ANC_mlw$prop_hiv28)) + sum(is.na(ANC_meiru_final$prop_hiv28)),
            sum(is.na(ANCsub_mlw$prop_hiv)) + sum(is.na(ANC_mlw$prop_hiv)) + sum(is.na(ANC_meiru_final$prop_hiv)),
            0,
            0)
  )

props_table$Outcomes <- factor(props_table$Outcomes, levels = c("Attended ANC < 28 weeks", "Tested for syphilis < 28 weeks","Tested for syphilis in pregnancy", "Tested for HIV < 28 weeks","Tested for HIV in pregnancy", "Received 1 dose of BPG", "Received 3 doses of BPG"))

props_table %>%
  knitr::kable(caption = "Coverage of WHO 95-95-95 process outcomes",col.names=c("Outcome Definition","Percent",  "Confidence Interval", "Number", "Total", "Not Known")) %>%
  kableExtra::kable_styling(full_width = FALSE)

## ----propstable-region-------------------------------------------------------------------------------------------------------
props_table_r <- data.frame(Outcomes = c("Attended ANC < 28 weeks", "Tested for syphilis < 28 weeks","Tested for syphilis in pregnancy", "Tested for HIV < 28 weeks","Tested for HIV in pregnancy", "Received 1 dose of BPG", "Received 3 doses of BPG"),
                            North = round (c(MLWprop_28_N, MLWprop_syph28_N, MLWprop_syph_N, MLWprop_hiv28_N,MLWprop_hiv_N, MLWprop_syphtrt_N, MLWprop_syphtrt3_N),2),
                            Central = round (c(MLWprop_28_C, MLWprop_syph28_C, MLWprop_syph_C,MLWprop_hiv28_C, MLWprop_hiv_C, MLWprop_syphtrt_C, MLWprop_syphtrt3_C),2),
                            South = round (c(MLWprop_28_S, MLWprop_syph28_S, MLWprop_syph_S, MLWprop_hiv28_S,MLWprop_hiv_S, MLWprop_syphtrt_S, MLWprop_syphtrt3_S),2))


props_table_r$Outcomes <- factor(props_table_r$Outcomes, levels = c("Attended ANC < 28 weeks", "Tested for syphilis < 28 weeks","Tested for syphilis in pregnancy", "Tested for HIV < 28 weeks","Tested for HIV in pregnancy", "Received 1 dose of BPG", "Received 3 doses of BPG"))

```

```r
props_table2_r <- props_table_r %>%
  pivot_longer(cols = c("North", "Central", "South"), names_to = "Region", values_to = "Percent")

props_table2_r <- props_table2_r %>%
  mutate(
    CI=c(paste(round(CI_MLWprop_28_N$conf.int[1] * 100,2),round(CI_MLWprop_28_N$conf.int[2]* 100, 2),sep = ", "),
         paste(round(CI_MLWprop_28_C$conf.int[1] * 100,2),round(CI_MLWprop_28_C$conf.int[2]* 100, 2),sep = ", "),
         paste(round(CI_MLWprop_28_S$conf.int[1] * 100,2),round(CI_MLWprop_28_S$conf.int[2]* 100, 2),sep = ", "),
         paste(round(CI_MLWprop_syph28_N$conf.int[1]* 100,2),round(CI_MLWprop_syph28_N$conf.int[2]* 100,2),sep = ", "),
         paste(round(CI_MLWprop_syph28_C$conf.int[1]* 100,2),round(CI_MLWprop_syph28_C$conf.int[2]* 100,2),sep = ", "),
         paste(round(CI_MLWprop_syph28_S$conf.int[1]* 100,2),round(CI_MLWprop_syph28_S$conf.int[2]* 100,2),sep = ", "),
         paste(round(CI_MLWprop_syph_N$conf.int[1]* 100,2),round(CI_MLWprop_syph_N$conf.int[2]* 100,2),sep = ", "),
         paste(round(CI_MLWprop_syph_C$conf.int[1]* 100,2),round(CI_MLWprop_syph_C$conf.int[2]* 100,2),sep = ", "),
         paste(round(CI_MLWprop_syph_S$conf.int[1]* 100,2),round(CI_MLWprop_syph_S$conf.int[2]* 100,2),sep = ", "),
         paste(round(CI_MLWprop_hiv28_N$conf.int[1]* 100,2),round(CI_MLWprop_hiv28_N$conf.int[2]* 100,2),sep = ", "),
         paste(round(CI_MLWprop_hiv28_C$conf.int[1]* 100,2),round(CI_MLWprop_hiv28_C$conf.int[2]* 100,2),sep = ", "),
         paste(round(CI_MLWprop_hiv28_S$conf.int[1]* 100,2),round(CI_MLWprop_hiv28_S$conf.int[2]* 100,2),sep = ", "),
         paste(round(CI_MLWprop_hiv_N$conf.int[1]* 100,2),round(CI_MLWprop_hiv_N$conf.int[2]* 100,2),sep = ", "),
         paste(round(CI_MLWprop_hiv_C$conf.int[1]* 100,2),round(CI_MLWprop_hiv_C$conf.int[2]* 100,2),sep = ", "),
         paste(round(CI_MLWprop_hiv_S$conf.int[1]* 100,2),round(CI_MLWprop_hiv_S$conf.int[2]* 100,2),sep = ", "),
         paste(round(CI_MLWprop_syphtrt_N$conf.int[1]* 100,2),round(CI_MLWprop_syphtrt_N$conf.int[2]* 100,2),sep = ", "),
         paste(round(CI_MLWprop_syphtrt_C$conf.int[1]* 100,2),round(CI_MLWprop_syphtrt_C$conf.int[2]* 100,2),sep = ", "),
         paste(round(CI_MLWprop_syphtrt_S$conf.int[1]* 100,2),round(CI_MLWprop_syphtrt_S$conf.int[2]* 100,2),sep = ", "),
         paste(round(CI_MLWprop_syphtrt3_N$conf.int[1]* 100,2),round(CI_MLWprop_syphtrt3_N$conf.int[2]* 100,2),sep = ", "),
         paste(round(CI_MLWprop_syphtrt3_C$conf.int[1]* 100,2),round(CI_MLWprop_syphtrt3_C$conf.int[2]* 100,2),sep = ", "),
         paste(round(CI_MLWprop_syphtrt3_S$conf.int[1]* 100,2),round(CI_MLWprop_syphtrt3_S$conf.int[2]* 100,2),sep = ", ")),

    number = c(sum(na.omit(ANC_mlw$prop_28_N)) + sum(na.omit(ANC_meiru_final$prop_28_N)),
               sum(na.omit(ANC_meiru_final$prop_28_C)),
               sum(na.omit(ANCsub_mlw$prop_28))+sum(na.omit(ANC_mlw$prop_28_S)) + sum(na.omit(ANC_meiru_final$prop_28_S)),
               sum(na.omit(filter(ANC_mlw, f_id==203)$prop_syph28)) + sum(na.omit(filter(ANC_meiru_final, f_id %in% c(202,203))$prop_syph28)),
               sum(na.omit(filter(ANC_meiru_final, f_id==201)$prop_syph28)),
               sum(na.omit(ANCsub_mlw$prop_syph28))+sum(na.omit(filter(ANC_mlw, f_id %in% 101:103)$prop_syph28)) + sum(na.omit(filter(ANC_meiru_final, f_id %in% 101:103)$prop_syph28)),
               sum(na.omit(filter(ANC_mlw, f_id==203)$prop_syph)) + sum(na.omit(filter(ANC_meiru_final, f_id %in% c(202,203))$prop_syph)),
               sum(na.omit(filter(ANC_meiru_final, f_id==201)$prop_syph)),
               sum(na.omit(ANCsub_mlw$prop_syph))+sum(na.omit(filter(ANC_mlw, f_id %in% 101:103)$prop_syph)) + sum(na.omit(filter(ANC_meiru_final, f_id %in% 101:103)$prop_syph)),
```

```
                    sum(na.omit(filter(ANC_mlw, f_id==203)$prop_hiv28)) + sum(na.omit(filter(ANC_meiru_final, f_id %in% c(202,203))$prop_hiv28)),
                    sum(na.omit(filter(ANC_meiru_final, f_id==201)$prop_hiv28)),
                    sum(na.omit(ANCsub_mlw$prop_hiv28))+sum(na.omit(filter(ANC_mlw, f_id %in% 101:103)$prop_hiv28)) + sum(na.omit(filter(ANC_meiru_final, f_id %in% 101:103)$prop_hiv28)),
                    sum(na.omit(filter(ANC_mlw, f_id==203)$prop_hiv)) + sum(na.omit(filter(ANC_meiru_final, f_id %in% c(202,203))$prop_hiv)),
                    sum(na.omit(filter(ANC_meiru_final, f_id==201)$prop_hiv)),
                    sum(na.omit(ANCsub_mlw$prop_hiv))+sum(na.omit(filter(ANC_mlw, f_id %in% 101:103)$prop_hiv)) + sum(na.omit(filter(ANC_meiru_final, f_id %in% 101:103)$prop_hiv)),
                    sum(na.omit(filter(DEL_meiru, f_id %in% c(202,203))$prop_syphtrt)),
                    sum(na.omit(filter(DEL_mlw, del_fid==201)$prop_syphtrt)) + sum(na.omit(filter(DEL_meiru, f_id==201)$prop_syphtrt)),
                    sum(na.omit(filter(DEL_mlw, del_fid %in% 101:104)$prop_syphtrt)),
                    sum(na.omit(filter(DEL_meiru, f_id %in% c(202,203))$prop_syphtrt3)),
                    sum(na.omit(filter(DEL_mlw, del_fid==201)$prop_syphtrt3)) + sum(na.omit(filter(DEL_meiru, f_id==201)$prop_syphtrt3)),
                    sum(na.omit(filter(DEL_mlw, del_fid %in% 101:104)$prop_syphtrt3))),

        total = c(nrow(filter(ANC_mlw, f_id==203)) + nrow(filter(ANC_meiru_final, f_id %in% c(202,203))),
                  nrow(filter(ANC_meiru_final, f_id==201)),
                  nrow(ANCsub_mlw) + nrow(filter(ANC_mlw, f_id %in% 101:103)) + nrow(filter(ANC_meiru_final, f_id %in% 101:103)),
                  nrow(filter(ANC_mlw, f_id==203)) + nrow(filter(ANC_meiru_final, f_id %in% c(202,203))),
                  nrow(filter(ANC_meiru_final, f_id==201)),
                  nrow(ANCsub_mlw) + nrow(filter(ANC_mlw, f_id %in% 101:103)) + nrow(filter(ANC_meiru_final, f_id %in% 101:103)),
                  nrow(filter(ANC_mlw, f_id==203)) + nrow(filter(ANC_meiru_final, f_id %in% c(202,203))),
                  nrow(filter(ANC_meiru_final, f_id==201)),
                  nrow(ANCsub_mlw) + nrow(filter(ANC_mlw, f_id %in% 101:103)) + nrow(filter(ANC_meiru_final, f_id %in% 101:103)),
                  nrow(filter(ANC_mlw, f_id==203)) + nrow(filter(ANC_meiru_final, f_id %in% c(202,203))),
                  nrow(filter(ANC_meiru_final, f_id==201)),
                  nrow(ANCsub_mlw) + nrow(filter(ANC_mlw, f_id %in% 101:103)) + nrow(filter(ANC_meiru_final, f_id %in% 101:103)),
                  nrow(filter(ANC_mlw, f_id==203)) + nrow(filter(ANC_meiru_final, f_id %in% c(202,203))),
                  nrow(filter(ANC_meiru_final, f_id==201)),
                  nrow(ANCsub_mlw) + nrow(filter(ANC_mlw, f_id %in% 101:103)) + nrow(filter(ANC_meiru_final, f_id %in% 101:103)),
                  sum(na.omit(filter(DEL_meiru, f_id %in% c(202,203))$del_result)),
                  sum(na.omit(filter(DEL_mlw, del_fid==201)$del_result)) + sum(na.omit(filter(DEL_meiru, f_id==201)$del_result)),
                  sum(na.omit(filter(DEL_mlw, del_fid %in% 101:104)$del_result)),
                  sum(na.omit(filter(DEL_meiru, f_id %in% c(202,203))$del_result)),
                  sum(na.omit(filter(DEL_mlw, del_fid==201)$del_result)) + sum(na.omit(filter(DEL_meiru, f_id==201)$del_result)),
                  sum(na.omit(filter(DEL_mlw, del_fid %in% 101:104)$del_result))),

        NAs = c(sum(is.na(ANC_mlw$prop_28_N)) + sum(is.na(ANC_meiru_final$prop_28_N)),
                sum(is.na(ANC_meiru_final$prop_28_C)),
                sum(is.na(ANCsub_mlw$prop_28))+sum(is.na(ANC_mlw$prop_28_S)) + sum(is.na(ANC_meiru_final$prop_28_S)),
                sum(is.na(filter(ANC_mlw, f_id==203)$prop_syph28)) + sum(is.na(filter(ANC_meiru_final, f_id %in% c(202,203))$prop_syph28)),
                sum(is.na(filter(ANC_meiru_final, f_id==201)$prop_syph28)),
                sum(is.na(ANCsub_mlw$prop_syph28))+sum(is.na(filter(ANC_mlw, f_id %in% 101:103)$prop_syph28)) + sum(is.na(filter(ANC_meiru_final, f_id %in% 101:103)$prop_syph28)),
                sum(is.na(filter(ANC_mlw, f_id==203)$prop_syph)) + sum(is.na(filter(
```

```
              ANC_meiru_final, f_id %in% c(202,203))$prop_syph)),
              sum(is.na(filter(ANC_meiru_final, f_id==201)$prop_syph)),
              sum(is.na(ANCsub_mlw$prop_syph))+sum(is.na(filter(ANC_mlw, f_id %in% 101:103
              )$prop_syph)) + sum(is.na(filter(ANC_meiru_final, f_id %in% 101:103)$
              prop_syph)),
              sum(is.na(filter(ANC_mlw, f_id==203)$prop_syph28)) + sum(is.na(filter(
              ANC_meiru_final, f_id %in% c(202,203))$prop_syph28)),
              sum(is.na(filter(ANC_meiru_final, f_id==201)$prop_syph28)),
              sum(is.na(ANCsub_mlw$prop_syph28))+sum(is.na(filter(ANC_mlw, f_id %in% 101:
              103)$prop_syph28)) + sum(is.na(filter(ANC_meiru_final, f_id %in% 101:103)$
              prop_syph28)),
              sum(is.na(filter(ANC_mlw, f_id==203)$prop_hiv)) + sum(is.na(filter(
              ANC_meiru_final, f_id %in% c(202,203))$prop_hiv)),
              sum(is.na(filter(ANC_meiru_final, f_id==201)$prop_hiv)),
              sum(is.na(ANCsub_mlw$prop_hiv))+sum(is.na(filter(ANC_mlw, f_id %in% 101:103)$
              prop_hiv)) + sum(is.na(filter(ANC_meiru_final, f_id %in% 101:103)$prop_hiv)),
              0,
              0,
              0,
              0,
              0,
              0)
   )



props_table2_r %>%
  knitr::kable(caption = "Coverage of WHO 95-95-95 process outcomes",col.names=c(
  "Outcome Definition","Region", "Percent",  "Confidence Interval", "Number", "Total",
  "Not known")) %>%
  kableExtra::kable_styling(full_width = FALSE)


##
----propsfig---------------------------------------------------------------------------
-----------------------------------

props_table %>%
  ggplot(mapping = aes(x=Outcomes, y=Percent)) +
  geom_bar(stat = "identity", position = "dodge", fill='#2596BE') +
  geom_text(aes(label = paste0(sprintf("%1.0f", Percent), "%")), position =
  position_dodge(width = .5), vjust = -0.4) +
  ggtitle("Coverage of WHO 95-95-95 process outcomes") +
  theme_classic()+
  theme(plot.title = element_text(hjust=0.5, size = 20), axis.title = element_text(size =
   12), axis.text.x = element_text(size = 10), axis.text.y = element_text(size = 10))+
  xlab("") +
  ylab("Percent (%)")+
  ylim(0,100)


props_table2_r %>%
  ggplot(mapping = aes(x=Outcomes, y=Percent, fill=Region)) +
  geom_bar(stat = "identity", position = "dodge") +
  geom_text(aes(label = paste0(sprintf("%1.0f", Percent), "%")), position =
  position_dodge(width = .9), vjust = -0.4) +
  scale_fill_brewer(palette = "Blues")+
  ggtitle("Coverage of WHO 95-95-95 process outcomes per region")+
  theme_classic() +
  theme(plot.title = element_text(hjust=0.5, size = 20), axis.title = element_text(size =
   12), axis.text.x = element_text(size = 9.5), axis.text.y = element_text(size = 10))+
  xlab("") +
  ylab("Percent (%)")+
  ylim(0,100)
```

```r
## ----bottleneckANC-target----------------------------------------------------------------------------------------------
#ANC data
#Target population

#combined sites
prop_all <- ((nrow(ANCsub_mlw) + nrow(ANC_mlw) + nrow(ANC_meiru_final)) /
               (nrow(ANCsub_mlw) + nrow(ANC_mlw) + nrow(ANC_meiru_final))) * 100




#By region
#North

prop_N <- ((nrow(ANC_mlw[ANC_mlw$f_id==203,]) + nrow(ANC_meiru_final[ANC_meiru_final$f_id %in% c(202,203),])) /
             (nrow(ANC_mlw[ANC_mlw$f_id==203,]) + nrow(ANC_meiru_final[ANC_meiru_final$f_id %in% c(202,203),]))) * 100

#Central
prop_C <- ((nrow(ANC_meiru_final[ANC_meiru_final$f_id==201,])) /
             (nrow(ANC_meiru_final[ANC_meiru_final$f_id==201,]))) * 100

#South
prop_S <- ((nrow(ANCsub_mlw) + nrow(ANC_mlw[ANC_mlw$f_id %in% 101:103,]) + nrow(ANC_meiru_final[ANC_meiru_final$f_id %in% 101:103,]))/
             (nrow(ANCsub_mlw) + nrow(ANC_mlw[ANC_mlw$f_id %in% 101:103,]) + nrow(ANC_meiru_final[ANC_meiru_final$f_id %in% 101:103,]))) * 100


## ----bottleneckANC-access----------------------------------------------------------------------------------------------
#Accessibility coverage


ANCsub_mlw <- ANCsub_mlw %>%
  mutate(
    prop_28_access = ifelse(((anc_ancvisit==1 | (is.na(anc_ancvisit))) & anc_gestationw < 28) | (anc_ancvisit>1 & diffwks_anc1 < 28), 1, 0)
  )

ANC_mlw <- ANC_mlw %>%
  mutate(
    prop_28_access = ifelse(gest_age_w < 28, 1, 0)
  )

ANC_meiru_final <- ANC_meiru_final %>%
  mutate(
    prop_28_access = ifelse(gest_age_w < 28, 1, 0)
  )


prop_28_access <- ((sum(na.omit(ANCsub_mlw$prop_28_access)) + sum(na.omit(ANC_mlw$prop_28_access)) + sum(na.omit(ANC_meiru_final$prop_28_access))) /
                     (nrow(ANCsub_mlw) + nrow(ANC_mlw) + nrow(ANC_meiru))) * 100



#North
prop_28_access_N <- ((sum(na.omit(filter(ANC_mlw, f_id==203)$prop_28_access)) + sum(na.omit(filter(ANC_meiru_final, f_id %in% c(202,203))$prop_28_access))) /
                       (nrow(ANC_mlw[ANC_mlw$f_id==203,]) + nrow(ANC_meiru_final[ANC_meiru_final$f_id %in% c(202,203),]))) * 100
```

```r


#Central
prop_28_access_C <- (sum(na.omit(filter(ANC_meiru_final, f_id==201)$prop_28_access)) /
                        (nrow(ANC_meiru_final[ANC_meiru_final$f_id==201,]))) * 100


#South
prop_28_access_S <- ((sum(na.omit(ANCsub_mlw$prop_28_access)) + sum(na.omit(filter(
ANC_mlw, f_id %in% 101:103)$prop_28_access)) + sum(na.omit(filter(ANC_meiru_final, f_id
%in% 101:103)$prop_28_access)))/
                        (nrow(ANCsub_mlw) + nrow(ANC_mlw[ANC_mlw$f_id %in% 101:103,]) +
                        nrow(ANC_meiru_final[ANC_meiru_final$f_id %in% 101:103,]))) * 100


##
----bottleneckANC-avail--------------------------------------------------------------------
------------------------------------


ANCsub_mlw <- ANCsub_mlw %>%
  mutate(
    prop_28_avail = ifelse(prop_28_access==1 & anc_syphdoc==1, 1, 0)
  )


ANC_mlw <- ANC_mlw %>%
  mutate(
    prop_28_avail = ifelse(prop_28_access==1 & syphillis==1, 1, 0)
  )

ANC_meiru_final <- ANC_meiru_final %>%
  mutate(
    prop_28_avail = ifelse(prop_28_access==1 & syphillis==1, 1, 0)
  )



prop_28_avail <- ((sum(na.omit(ANCsub_mlw$prop_28_avail)) + sum(na.omit(ANC_mlw$
prop_28_avail)) + sum(na.omit(ANC_meiru_final$prop_28_avail))) /
                     (sum(na.omit(ANCsub_mlw$prop_28_access)) + sum(na.omit(ANC_mlw$
                     prop_28_access)) + sum(na.omit(ANC_meiru_final$prop_28_access)))) *
                     100


#North
prop_28_avail_N <- ((sum(na.omit(filter(ANC_mlw, f_id==203)$prop_28_avail)) + sum(na.omit
(filter(ANC_meiru_final, f_id %in% c(202,203))$prop_28_avail))) /
                      (sum(na.omit(filter(ANC_mlw, f_id==203)$prop_28_access)) + sum(
                      na.omit(filter(ANC_meiru_final, f_id %in% c(202,203))$
                      prop_28_access)))) * 100


#Central
prop_28_avail_C <- (sum(na.omit(filter(ANC_meiru_final, f_id==201)$prop_28_avail)) /
                      sum(na.omit(filter(ANC_meiru_final, f_id==201)$prop_28_access))) *
                      100


#South
prop_28_avail_S <- ((sum(na.omit(ANCsub_mlw$prop_28_avail)) + sum(na.omit(filter(ANC_mlw
, f_id %in% 101:103)$prop_28_avail)) + sum(na.omit(filter(ANC_meiru_final, f_id %in% 101:
103)$prop_28_avail)))/
                      (sum(na.omit(ANCsub_mlw$prop_28_access)) + sum(na.omit(filter(
                      ANC_mlw, f_id %in% 101:103)$prop_28_access)) + sum(na.omit(filter(
                      ANC_meiru_final, f_id %in% 101:103)$prop_28_access)))) * 100
```

```r
## ----bottleneckANC-effect-----------------------------------------------------------------------------------------------------

ANCsub_mlw <- ANCsub_mlw %>%
  mutate(
    anc_inj1dt=dmy(anc_inj1dt),
    anc_prevpos=dmy(anc_prevpos),
    prop_28_effect = ifelse(prop_28_access==1 & anc_syphdoc==1 & anc_pos==1 & anc_syphtest==1 & anc_dtprev==1 & anc_inj1doc==1 & anc_inj1dt==anc_prevpos, 1, 0)
  )


ANC_mlw <- ANC_mlw %>%
  mutate(
    prop_28_effect = ifelse(prop_28_access==1 & syphillis==1 & resultsy==1 & benza==1, 1, 0)
  )

ANC_meiru_final <- ANC_meiru_final %>%
  mutate(
    prop_28_effect = ifelse(prop_28_access==1 & syphillis==1 & resultsy==1 & benza==1, 1, 0)
  )



prop_28_effect <- ((sum(na.omit(ANCsub_mlw$prop_28_effect)) + sum(na.omit(ANC_mlw$prop_28_effect)) + sum(na.omit(ANC_meiru_final$prop_28_effect))) /
                     (sum(na.omit(ANCsub_mlw$anc_pos)) + sum(na.omit(ANC_mlw$resultsy)) + sum(na.omit(ANC_meiru_final$resultsy)))) * 100

#North
prop_28_effect_N <- ((sum(na.omit(filter(ANC_mlw, f_id==203)$prop_28_effect)) + sum(na.omit(filter(ANC_meiru_final, f_id %in% c(202,203))$prop_28_effect))) /
                       (sum(na.omit(filter(ANC_mlw, f_id==203)$resultsy)) + sum(na.omit(filter(ANC_meiru_final, f_id %in% c(202,203))$resultsy)))) * 100


#Central
prop_28_effect_C <- (sum(na.omit(filter(ANC_meiru_final, f_id==201)$prop_28_effect)) /
                       sum(na.omit(filter(ANC_meiru_final, f_id==201)$resultsy))) * 100


#South
prop_28_effect_S <- ((sum(na.omit(ANCsub_mlw$prop_28_effect)) + sum(na.omit(filter(ANC_mlw, f_id %in% 101:103)$prop_28_effect)) + sum(na.omit(filter(ANC_meiru_final, f_id %in% 101:103)$prop_28_effect)))/
                       (sum(na.omit(ANCsub_mlw$anc_pos)) + sum(na.omit(filter(ANC_mlw, f_id %in% 101:103)$resultsy)) + sum(na.omit(filter(ANC_meiru_final, f_id %in% 101:103)$resultsy)))) * 100


## ----bottleneckANC-figs-------------------------------------------------------------------------------------------------------
#table of proportions (%)

bottleneck_1 <- data.frame(Outcomes = c("Target population", "Accessibility coverage", "Availability coverage", "Effective coverage"),
                           Percent = c(prop_all, prop_28_access, prop_28_avail, prop_28_effect))

bottleneck_1$Outcomes <- factor(bottleneck_1$Outcomes, levels = c("Target population",
```

```
"Accessibility coverage", "Availability coverage", "Effective coverage"))

bottleneck_1_r <- data.frame(Outcomes = c("Target population", "Accessibility coverage",
"Availability coverage", "Effective coverage"),
                            North = c(prop_N, prop_28_access_N, prop_28_avail_N,
                            prop_28_effect_N),
                            Central = c(prop_C, prop_28_access_C, prop_28_avail_C,
                            prop_28_effect_C),
                            South = c(prop_S, prop_28_access_S, prop_28_avail_S,
                            prop_28_effect_S))

bottleneck_1_r$Outcomes <- factor(bottleneck_1_r$Outcomes, levels = c("Target population"
, "Accessibility coverage", "Availability coverage", "Effective coverage"))

bottleneck_1_r$Denom <- c("Total", "Total", "Total", "Positive")

bottleneck_1_r2 <- bottleneck_1_r %>%
  pivot_longer(cols = c("North", "Central", "South"), names_to = "Region", values_to =
  "Percent")


bottleneck_1 %>%
  ggplot(mapping = aes(x=Outcomes, y=Percent, width=.7, fill=Outcomes)) +
  geom_bar(stat = "identity", position = "identity") +
  scale_fill_manual(values= c('#2badab','#2badab', '#2badab', '#2596BE')) +
  geom_text(aes(label = paste0(sprintf("%1.0f", Percent), "%")),size=2, hjust = -0.8,
  nudge_y = -2.2) +
  coord_flip() +
  scale_x_discrete(limits=rev(levels((bottleneck_1$Outcomes)))) +
  theme_classic()+
  ylab("Percent (%)") +
  xlab("") +
  guides(fill=FALSE)


bottleneck_1_r2 %>%
  ggplot(mapping = aes(x=reorder(Outcomes, +Percent), y=Percent, fill=Region, width=.8))
  +
  geom_bar(stat = "identity", position = position_dodge(.8)) +
  geom_text(aes(label = paste0(sprintf("%1.0f", Percent), "%")), size=2, position =
  position_dodge(width = .9), hjust = -0.05) +
  scale_fill_brewer(palette = "Blues")+
  coord_flip() +
  scale_x_discrete(limits=rev(levels((bottleneck_1_r2$Outcomes)))) +
  theme_classic()+
  xlab("") +
  ylab("Percent (%)")+
  guides(fill = guide_legend(reverse = TRUE))


bottleneck_1_r2 %>%
  dplyr::mutate(Denom=case_when(Denom=="Total"~"All",Denom=="Positive"~"Positive")) %>%
  dplyr::mutate(RegionDenom=factor(paste(sep=" - ",Region,Denom),levels=c("North -
  Positive","Central - Positive","South - Positive","North - All","Central - All","South
  - All"))) %>%
  ggplot(mapping = aes(x=Outcomes, y=Percent, width=.8)) +
  geom_bar(stat = "identity", position = position_dodge(.8), aes(fill=RegionDenom, alpha
  = RegionDenom)) +
  scale_fill_manual(values= c(rep('#2596BE',3), rep('#2badab',3)),labels=c("North -
  Positive","Central - Positive","South - Positive","North - All","Central - All","South
  - All"),name="Region & population") +
  geom_text(aes(label = paste0(sprintf("%1.0f", Percent), "%"), group=RegionDenom), size=
  2, position = position_dodge(width = .9), hjust = -0.15) +
  scale_alpha_manual(values = rep(c(0.3, 0.6, 0.9),2),labels=c("North - Positive",
  "Central - Positive","South - Positive","North - All","Central - All","South - All"),
  name="Region & population") +
```

```r
  coord_flip() +
  scale_x_discrete(limits=rev(levels((bottleneck_1_r2$Outcomes)))) +
  theme_classic()+
  xlab("") +
  ylab("Percent (%)")




## ----bottleneckDEL-target------------------------------------------------------------------------------------------------
#target population

#combined sites
prop_all_d <- (nrow(DEL_mlw) + nrow(DEL_meiru)) /
  (nrow(DEL_mlw) + nrow(DEL_meiru)) * 100


#By region
#North




prop_d_N <- (nrow(DEL_meiru[DEL_meiru$f_id %in% c(202,203),]) /
               nrow(DEL_meiru[DEL_meiru$f_id %in% c(202,203),]))  * 100

#Central
prop_d_C <- ((nrow(DEL_mlw[DEL_mlw$del_fid==201,]) + nrow(DEL_meiru[DEL_meiru$f_id==201,])) /
               (nrow(DEL_mlw[DEL_mlw$del_fid==201,]) + nrow(DEL_meiru[DEL_meiru$f_id==201,]))) * 100

#South
prop_d_S <- (nrow(DEL_mlw[DEL_mlw$del_fid%in% 101:104,])  /
               nrow(DEL_mlw[DEL_mlw$del_fid%in% 101:104,])) * 100


## ----bottleneckDEL-access------------------------------------------------------------------------------------------------

DEL_mlw <- DEL_mlw %>%
  mutate(
    access_d = ifelse(del_syphilis==1 | del_testtoday==1, 1, 0)
  )

DEL_meiru <- DEL_meiru %>%
  mutate(
    access_d = ifelse(del_syph==1 | del_testtoday==1, 1, 0)
  )

#total
prop_access_d <-  ((sum(na.omit(DEL_mlw$access_d)) + sum(na.omit(DEL_meiru$access_d)))/
                     (nrow(DEL_mlw) + nrow(DEL_meiru)))* 100

#By region
#North

prop_access_d_N <- (sum(na.omit(filter(DEL_meiru, f_id %in% c(202,203))$access_d)) /
                      nrow(DEL_meiru[DEL_meiru$f_id %in% c(202,203),])) * 100

#Central
prop_access_d_C <- ((sum(na.omit(filter(DEL_mlw, del_fid==201)$access_d)) + sum(na.omit(filter(DEL_meiru, f_id==201)$access_d))) /
```

```r
                     (nrow(DEL_mlw[DEL_mlw$del_fid==201,]) + nrow(DEL_meiru[DEL_meiru$f_id==201,]))) * 100

#South
prop_access_d_S <- (sum(na.omit(filter(DEL_mlw, del_fid %in% 101:104)$access_d)) /
                      nrow(DEL_mlw[DEL_mlw$del_fid%in% 101:104,])) * 100



## ----bottleneckDEL-avail---------------------------------------------------------------------------------------------
DEL_mlw <- DEL_mlw %>%
  mutate(
    prop_avail_d = ifelse(del_syphilis == 1 & del_result==1 & del_treatment==1 & del_injections %in% 1:3, 1, 0)
  )

DEL_meiru <- DEL_meiru %>%
  mutate(
    prop_avail_d = ifelse(del_syph == 1 & del_result==1 & del_rx==1 &del_treat %in% 1:3, 1, 0)
  )




prop_avail_d <- ((sum(na.omit(DEL_mlw$prop_avail_d)) + sum(na.omit(DEL_meiru$prop_avail_d)))/
                   (sum(na.omit(DEL_mlw$del_result)) + sum(na.omit(DEL_meiru$del_result))))* 100

#By region
#North

prop_avail_d_N <- (sum(na.omit(filter(DEL_meiru, f_id %in% c(202,203))$prop_avail_d)) /
                     sum(na.omit(filter(DEL_meiru, f_id %in% c(202,203))$del_result))) * 100

#Central
prop_avail_d_C <- ((sum(na.omit(filter(DEL_mlw, del_fid==201)$prop_avail_d)) + sum(na.omit(filter(DEL_meiru, f_id==201)$prop_avail_d))) /
                     (sum(na.omit(filter(DEL_mlw, del_fid==201)$del_result)) + sum(na.omit(filter(DEL_meiru, f_id==201)$del_result)))) * 100

#South
prop_avail_d_S <- (sum(na.omit(filter(DEL_mlw, del_fid %in% 101:104)$prop_avail_d)) /
                     sum(na.omit(filter(DEL_mlw, del_fid %in% 101:104)$del_result))) * 100



## ----bottleneckDEL-effect1-------------------------------------------------------------------------------------------
DEL_mlw <- DEL_mlw %>%
  mutate(
    prop_effect_d = ifelse((del_result==1 & del_treatment==1 & del_injections %in% 1:3 & (del_inj1doc==1 | del_inj2doc==1 | del_inj3doc==1) & (diffDate_inj1dt>=30 | diffDate_inj2dt>=30 | diffDate_inj3dt>=30)) |
                             (del_result==1 & del_treatment==1 & (del_injections==9 | (del_injections %in% 1:3 & (del_inj1doc==0 | del_inj2doc==0 | del_inj3doc==0)) | (del_injections%in% 1:3 & (del_inj1doc==1 | del_inj2doc==1 | del_inj3doc==1)& (diffDate_inj1dt<30 | diffDate_inj2dt<30 | diffDate_inj3dt<30))) & del_recommend==1), 1, 0)
```

```r
  )

DEL_meiru <- DEL_meiru %>%
  mutate(
    prop_effect_d = ifelse((del_result==1 & del_rx==1 & del_treat %in% 1:3 & (del_dt1doc==1 | del_dt2doc==1 | del_dt3doc==1) & (diffDate_dt1>=30 | diffDate_dt2>=30 | diffDate_dt3>=30)) |
                             (del_result==1 & del_rx==1 & ((del_treat %in% 1:3 & (del_dt1doc==0 | del_dt2doc==0 | del_dt3doc==0)) | (del_treat %in% 1:3 & (del_dt1doc==1 | del_dt2doc==1 | del_dt3doc==1) & (diffDate_dt1<30 | diffDate_dt2<30 | diffDate_dt3<30))) & del_rxkid==1), 1, 0)
  )


prop_effect_d <- ((sum(na.omit(DEL_mlw$prop_effect_d)) + sum(na.omit(DEL_meiru$prop_effect_d)))/
                    (sum(na.omit(DEL_mlw$del_result)) + sum(na.omit(DEL_meiru$del_result))))* 100

#By region
#North

prop_effect_d_N <- (sum(na.omit(filter(DEL_meiru, f_id %in% c(202,203))$prop_effect_d)) /
                      sum(na.omit(filter(DEL_meiru, f_id %in% c(202,203))$del_result))) * 100

#Central
prop_effect_d_C <- ((sum(na.omit(filter(DEL_mlw, del_fid==201)$prop_effect_d)) + sum(na.omit(filter(DEL_meiru, f_id==201)$prop_effect_d))) /
                      (sum(na.omit(filter(DEL_mlw, del_fid==201)$del_result)) + sum(na.omit(filter(DEL_meiru, f_id==201)$del_result)))) * 100

#South
prop_effect_d_S <- (sum(na.omit(filter(DEL_mlw, del_fid %in% 101:104)$prop_effect_d)) /
                      sum(na.omit(filter(DEL_mlw, del_fid %in% 101:104)$del_result))) * 100


## ----bottleneckDEL-effect2----------------------------------------------------------------------------------------------------
DEL_mlw <- DEL_mlw %>%
  mutate(
    prop_effect_d_b = ifelse((del_result==1 & del_treatment==1 & del_injections==3 & del_inj3doc==1 & diffDate_inj3dt>=30) |
                               (del_result==1 & del_treatment==1 & (del_injections %in% 1:2 | (del_injections==3 & del_inj3doc==0 ) | (del_injections==3 & del_inj3doc==1 & diffDate_inj3dt<30)) & del_recommend==1), 1, 0)
  )

DEL_meiru <- DEL_meiru %>%
  mutate(
    prop_effect_d_b = ifelse((del_result==1 & del_rx==1 & del_treat==3 & del_dt3doc==1 & diffDate_dt3>=30) |
                               (del_result==1 & del_rx==1 & (del_treat %in% 1:2 | (del_treat==3 & del_dt3doc==0) | (del_treat==3 & del_dt3doc==1 & diffDate_dt3<30)) & del_rxkid==1), 1, 0)
  )


prop_effect_d_b <- ((sum(na.omit(DEL_mlw$prop_effect_d_b)) + sum(na.omit(DEL_meiru$prop_effect_d_b)))/
                      (sum(na.omit(DEL_mlw$del_result)) + sum(na.omit(DEL_meiru$del_result))))* 100
```

```r

#By region
#North

prop_effect_d_b_N <- (sum(na.omit(filter(DEL_meiru, f_id %in% c(202,203))$prop_effect_d_b)) /
                            sum(na.omit(filter(DEL_meiru, f_id %in% c(202,203))$del_result))) * 100

#Central
prop_effect_d_b_C <- ((sum(na.omit(filter(DEL_mlw, del_fid==201)$prop_effect_d_b)) + sum(na.omit(filter(DEL_meiru, f_id==201)$prop_effect_d_b))) /
                            (sum(na.omit(filter(DEL_mlw, del_fid==201)$del_result)) + sum(na.omit(filter(DEL_meiru, f_id==201)$del_result)))) * 100

#South
prop_effect_d_b_S <- (sum(na.omit(filter(DEL_mlw, del_fid %in% 101:104)$prop_effect_d_b)) /
                            sum(na.omit(filter(DEL_mlw, del_fid %in% 101:104)$del_result))) * 100


## ----bottleneckDEL-figs-----------------------------------------------------------------------------------------
#table of proportions (%)

bottleneck_2 <- data.frame(Outcomes = c("Target population", "Accessibility coverage", "Availability coverage", "Effective coverage - WHO", "Effective coverage - MLW"),
                           Percent = c(prop_all_d, prop_access_d, prop_avail_d, prop_effect_d, prop_effect_d_b))

bottleneck_2$Outcomes <- factor(bottleneck_2$Outcomes, levels = c("Target population", "Accessibility coverage", "Availability coverage", "Effective coverage - WHO", "Effective coverage - MLW"))


bottleneck_2_r <- data.frame(Outcomes = c("Target population", "Accessibility coverage", "Availability coverage", "Effective coverage - WHO", "Effective coverage - MLW"),
                             North = c(prop_d_N, prop_access_d_N, prop_avail_d_N, prop_effect_d_N, prop_effect_d_b_N),
                             Central = c(prop_d_C, prop_access_d_C, prop_avail_d_C, prop_effect_d_C, prop_effect_d_b_C),
                             South = c(prop_d_S, prop_access_d_S, prop_avail_d_S, prop_effect_d_S, prop_effect_d_b_S))

bottleneck_2_r$Outcomes <- factor(bottleneck_2_r$Outcomes, levels = c("Target population", "Accessibility coverage", "Availability coverage", "Effective coverage - WHO", "Effective coverage - MLW"))


bottleneck_2_r$Denom <- c("Total", "Total", "Positive", "Positive", "Positive")


bottleneck_2_r2 <- bottleneck_2_r %>%
  pivot_longer(cols = c("North", "Central", "South"), names_to = "Region", values_to = "Percent")
#################################################################################
bottleneck_2 %>%
  ggplot(mapping = aes(x=Outcomes, y=Percent, width=.7, fill=Outcomes)) +
  geom_bar(stat = "identity", position = "identity") +
  scale_fill_manual(values= c('#2badab', '#2badab', '#2596BE','#2596BE', '#2596BE' )) +
  geom_text(aes(label = paste0(sprintf("%1.0f", Percent), "%")),size=2, hjust = -0.68, nudge_y = -2) +
  coord_flip() +
  scale_x_discrete(limits=rev(levels((bottleneck_2$Outcomes)))) +
```

```r
  theme_classic()+
  ylab("Percent (%)") +
  xlab("") +
  theme(legend.position = "none")



bottleneck_2_r2 %>%
  dplyr::mutate(Denom=case_when(Denom=="Total"~"All",Denom=="Positive"~"Positive")) %>%
  dplyr::mutate(RegionDenom=factor(paste(sep=" - ",Region,Denom),levels=c("North - Positive","Central - Positive","South - Positive","North - All","Central - All","South - All"))) %>%
  ggplot(mapping = aes(x=Outcomes, y=Percent, width=.8)) +
  geom_bar(stat = "identity", position = position_dodge(.8), aes(fill=RegionDenom, alpha = RegionDenom)) +
  scale_fill_manual(values= c(rep('#2596BE',3), rep('#2badab',3)),labels=c("North - Positive","Central - Positive","South - Positive","North - All","Central - All","South - All"),name="Region & population") +
  geom_text(aes(label = paste0(sprintf("%1.0f", Percent), "%"), group=RegionDenom), size=2, position = position_dodge(width = .9), hjust = -0.01) +
  scale_alpha_manual(values = rep(c(0.3, 0.6, 0.9),2),labels=c("North - Positive", "Central - Positive","South - Positive","North - All","Central - All","South - All"), name="Region & population") +
  coord_flip() +
  scale_x_discrete(limits=rev(levels((bottleneck_2_r2$Outcomes)))) +
  theme_classic()+
  xlab("") +
  ylab("Percent (%)")



## ----nottreated---------------------------------------------------------------------------------------------------------------

DEL_mlw <- DEL_mlw %>%
  mutate(
    nottreat_30 = ifelse(del_result==1 & del_treatment==1 & (del_injections==9 | (del_injections %in% 1:3 & (del_inj1doc==0 | del_inj2doc==0 | del_inj3doc==0)) | (del_injections%in% 1:3 & (del_inj1doc==1 | del_inj2doc==1 | del_inj3doc==1)& (diffDate_inj1dt<30 | diffDate_inj2dt<30 | diffDate_inj3dt<30))), 1, 0),
    nottreat_30_inf = ifelse(del_result==1 & del_treatment==1 & (del_injections==9 | (del_injections %in% 1:3 & (del_inj1doc==0 | del_inj2doc==0 | del_inj3doc==0)) | (del_injections%in% 1:3 & (del_inj1doc==1 | del_inj2doc==1 | del_inj3doc==1)& (diffDate_inj1dt<30 | diffDate_inj2dt<30 | diffDate_inj3dt<30))) & del_recommend==1, 1, 0),
    nottreat_30_b = ifelse(del_result==1 & del_treatment==1 & (del_injections %in% 1:2 | (del_injections==3 & del_inj3doc==0 ) | (del_injections==3 & del_inj3doc==1 & diffDate_inj3dt<30)), 1, 0),
    nottreat_30_inf_b = ifelse(del_result==1 & del_treatment==1 & (del_injections %in% 1:2 | (del_injections==3 & del_inj3doc==0 ) | (del_injections==3 & del_inj3doc==1 & diffDate_inj3dt<30)) & del_recommend==1, 1, 0)
  )


DEL_meiru <- DEL_meiru %>%
  mutate(
    nottreat_30 = ifelse(del_result==1 & del_rx==1 & ((del_treat %in% 1:3 & (del_dt1doc==0 | del_dt2doc==0 | del_dt3doc==0)) | (del_treat %in% 1:3 & (del_dt1doc==1 | del_dt2doc==1 | del_dt3doc==1) & (diffDate_dt1<30 | diffDate_dt2<30 | diffDate_dt3<30))), 1, 0),
    nottreat_30_inf = ifelse(del_result==1 & del_rx==1 & ((del_treat %in% 1:3 & (del_dt1doc==0 | del_dt2doc==0 | del_dt3doc==0)) | (del_treat %in% 1:3 & (del_dt1doc==1 | del_dt2doc==1 | del_dt3doc==1) & (diffDate_dt1<30 | diffDate_dt2<30 | diffDate_dt3<30))) & del_rxkid==1, 1, 0),
    nottreat_30_b = ifelse(del_result==1 & del_rx==1 & (del_treat %in% 1:2 | (del_treat==
```

```
    3 & del_dt3doc==0) | (del_treat==3 & del_dt3doc==1 & diffDate_dt3<30)), 1, 0),
    nottreat_30_inf_b = ifelse(del_result==1 & del_rx==1 & (del_treat %in% 1:2 | (
    del_treat==3 & del_dt3doc==0) | (del_treat==3 & del_dt3doc==1 & diffDate_dt3<30)) &
    del_rxkid==1, 1, 0)
  )

#women who were treated with atleast one dose of treatment <30 days

nottreat_1dose = sum(na.omit(DEL_mlw$nottreat_30)) + sum(na.omit(DEL_meiru$nottreat_30))
#number that received/referred for infant treatment
nottreat_1dose_inf = sum(na.omit(DEL_mlw$nottreat_30_inf)) + sum(na.omit(DEL_meiru$
nottreat_30_inf))

#women who were treated with three doses of treatment <30 days

nottreat_3dose = sum(na.omit(DEL_mlw$nottreat_30_b)) + sum(na.omit(DEL_meiru$
nottreat_30_b))
#number that received/referred for infant treatment
nottreat_3dose_inf = sum(na.omit(DEL_mlw$nottreat_30_inf_b)) + sum(na.omit(DEL_meiru$
nottreat_30_inf_b))

#-----------------------------------------------------------------------------------------
---------------------------------------------------

## Objective 4

##
----loaddata------------------------------------------------------------------------------
-----------------------------------

#Load MLW data

DEL_mlw <- read_dta("SyphStat_Delivery.dta")
DEL_meiru <- read_dta("Syph_Delivery_Meiru.dta")



##
----WHO-CS--------------------------------------------------------------------------------
-----------------------------------
#for each individual centre
#MLW data

##### create time difference variables

DEL_mlw<-DEL_mlw %>%
  dplyr::mutate(
    data_date=as.Date(data_date),
    del_inj1dt=lubridate::dmy(del_inj1dt),
    del_inj2dt=lubridate::dmy(del_inj2dt),
    del_inj3dt=lubridate::dmy(del_inj3dt),
  ) %>%
  dplyr::mutate(
    diffDate_inj1dt=data_date-del_inj1dt,
    diffDate_inj2dt=data_date-del_inj2dt,
    diffDate_inj3dt=data_date-del_inj3dt
  )


DEL_mlw$mindt <- apply(DEL_mlw[, c("del_inj1dt", "del_inj2dt", "del_inj3dt")], 1,
function(x) min(x, na.rm = TRUE))
DEL_mlw$mindt <- as.Date(DEL_mlw$mindt)
DEL_mlw$diffDate <- DEL_mlw$data_date - DEL_mlw$mindt


DEL_mlw <- DEL_mlw %>%
  mutate(
```

```
    Inc_CS_who=ifelse((del_syphilis==1 & del_result==1 & del_treatment %in% c(0,9)) |
                        (del_syphilis==1 & del_result==1 & del_treatment==1 &
                        del_injections %in% c(1:3,9) & (del_inj1doc==0 | del_inj2doc==0 |
                         del_inj3doc==0)) |
                        ( del_syphilis==1 & del_result==1 & del_treatment==1 &
                        del_injections %in% 1:3 & (del_inj1doc==1 | del_inj2doc==1 |
                        del_inj3doc==1) & diffDate<30), 1, 0),
    denom_CS=ifelse(del_syphilis==1 & del_live==1, 1, 0)
  )




#Meiru data

DEL_meiru<-DEL_meiru %>%
  dplyr::mutate(
    data_date=as.Date(data_date),
    del_dt1=lubridate::dmy(del_dt1),
    del_dt2=lubridate::dmy(del_dt2),
    del_dt3=lubridate::dmy(del_dt3),
  ) %>%
  dplyr::mutate(
    diffDate_dt1=data_date-del_dt1,
    diffDate_dt2=data_date-del_dt2,
    diffDate_dt3=data_date-del_dt3
  )


DEL_meiru$mindt <- apply(DEL_meiru[, c("del_dt1", "del_dt2", "del_dt3")], 1, function(x)
min(x, na.rm = TRUE))
DEL_meiru$mindt <- ymd(DEL_meiru$mindt)
DEL_meiru$diffDate <- DEL_meiru$data_date - DEL_meiru$mindt

DEL_meiru <- DEL_meiru %>%
  mutate(
    Inc_CS_who=ifelse((del_syph==1 & del_result==1 & del_rx==0)  |
                        (del_syph==1 & del_result==1 & del_rx==1 & del_treat %in% 1:3 & (
                        del_dt1doc==0 | del_dt2doc==0 | del_dt3doc==0)) |
                        (del_syph==1 & del_result==1 & del_rx==1 & del_treat %in% 1:3 & (
                        del_dt1doc==1 | del_dt2doc==1 | del_dt3doc==1) & diffDate<30), 1
                        , 0),
    denom_CS=ifelse(del_syph==1 & (DEL_meiru$livebirth==1 | DEL_meiru$livebirth2==1 |
    DEL_meiru$livebirth3==1),1,0)
  )

Inc_CS_who_mlw <- (sum(DEL_mlw$Inc_CS_who, na.rm = TRUE) + sum(DEL_meiru$Inc_CS_who,
na.rm = TRUE))/ (sum(DEL_mlw$denom_CS, na.rm = TRUE) + sum(DEL_meiru$denom_CS, na.rm =
TRUE)) * 100000



##########################################################################################
############################

#By region
#North, Central, South

#No data from the North in MLW
#one from the central
#the rest are from the south
DEL_mlw <- DEL_mlw %>%
  mutate(
    Inc_CS_who_C=ifelse(del_fid==201 & ((del_syphilis==1 & del_result==1 & del_treatment
    %in% c(0,9)) |
                                            (del_syphilis==1 & del_result==1 &
```

```
                                                    del_treatment==1 & del_injections %in% c(1:3,9) & (del_inj1doc==0 | del_inj2doc==0 | del_inj3doc==0)) |
                                                    ( del_syphilis==1 & del_result==1 & del_treatment==1 & del_injections %in% 1:3 & (del_inj1doc==1 | del_inj2doc==1 | del_inj3doc==1) & diffDate<30)), 1, 0),
    Inc_CS_who_S=ifelse(del_fid %in% 101:104 & ((del_syphilis==1 & del_result==1 & del_treatment %in% c(0,9)) |
                                                    (del_syphilis==1 & del_result==1 & del_treatment==1 & del_injections %in% c(1:3,9) & (del_inj1doc==0 | del_inj2doc==0 | del_inj3doc==0)) |
                                                    ( del_syphilis==1 & del_result==1 & del_treatment==1 & del_injections %in% 1:3 & (del_inj1doc==1 | del_inj2doc==1 | del_inj3doc==1) & diffDate<30)), 1, 0 )
  )

#CS is 0 in the central in the mlw data

Inc_CS_who_mlw_S <- sum(DEL_mlw$Inc_CS_who_S, na.rm = TRUE) / sum(filter(DEL_mlw, del_fid %in% 101:104)$denom_CS, na.rm = TRUE) * 100000


#No data from the South in MEIRU data
DEL_meiru <- DEL_meiru %>%
  mutate(
    Inc_CS_who_N=ifelse(f_id %in% 202:203 & ((del_syph==1 & del_result==1 & del_rx==0)  |
                                              (del_syph==1 & del_result==1 & del_rx==1 & del_treat %in% 1:3 & (del_dt1doc==0 | del_dt2doc==0 | del_dt3doc==0)) |
                                              (del_syph==1 & del_result==1 & del_rx==1 & del_treat %in% 1:3 & (del_dt1doc==1 | del_dt2doc==1 | del_dt3doc==1) & diffDate<30)), 1, 0),
    Inc_CS_who_C=ifelse(f_id==201 & ((del_syph==1 & del_result==1 & del_rx==0)  |
                                      (del_syph==1 & del_result==1 & del_rx==1 & del_treat %in% 1:3 & (del_dt1doc==0 | del_dt2doc==0 | del_dt3doc==0)) |
                                      (del_syph==1 & del_result==1 & del_rx==1 & del_treat %in% 1:3 & (del_dt1doc==1 | del_dt2doc==1 | del_dt3doc==1) & diffDate<30)), 1, 0)
  )

#no CS data from the north and south in the meiru data

Inc_CS_who_meiru_C <- sum(DEL_meiru$Inc_CS_who_C, na.rm = TRUE) / sum(filter(DEL_meiru, f_id == 201)$denom_CS, na.rm = TRUE) * 100000

###########################################################################
#By urban vs rural
DEL_mlw <- DEL_mlw %>%
  mutate(
    Inc_CS_who_U=ifelse(del_fid %in% c(101:104, 201) & ((del_syphilis==1 & del_result==1 & del_treatment %in% c(0,9)) |
                                                          (del_syphilis==1 & del_result==1 & del_treatment==1 & del_injections %in% c(1:3,9) & (del_inj1doc==0 | del_inj2doc==0 | del_inj3doc==0)) |
                                                          ( del_syphilis==1 & del_result==1 & del_treatment==1 & del_injections %in% 1:3 & ( del_inj1doc==1 | del_inj2doc==1
```

```
                                                               | del_inj3doc==1) & diffDate<30)), 1, 0)
  )

Inc_CS_who_mlw_U <- sum(DEL_mlw$Inc_CS_who_U, na.rm = TRUE) / sum(filter(DEL_mlw, del_fid %in% c(101:201, 203))$denom_CS, na.rm = TRUE) * 100000



DEL_meiru <- DEL_meiru %>%
  mutate(
    Inc_CS_who_U=ifelse(f_id %in% c(201, 203) & ((del_syph==1 & del_result==1 & del_rx==0)  |
                                                   (del_syph==1 & del_result==1 & del_rx==1 & del_treat %in% 1:3 & (del_dt1doc==0 | del_dt2doc==0 | del_dt3doc==0)) |
                                                   (del_syph==1 & del_result==1 & del_rx==1 & del_treat %in% 1:3 & (del_dt1doc==1 | del_dt2doc==1 | del_dt3doc==1) & diffDate<30)), 1, 0),
    Inc_CS_who_R=ifelse(f_id==202 & ((del_syph==1 & del_result==1 & del_rx==0)  |
                                     (del_syph==1 & del_result==1 & del_rx==1 & del_treat %in% 1:3 & (del_dt1doc==0 | del_dt2doc==0 | del_dt3doc==0)) |
                                     (del_syph==1 & del_result==1 & del_rx==1 & del_treat %in% 1:3 & (del_dt1doc==1 | del_dt2doc==1 | del_dt3doc==1) & diffDate<30)), 1, 0)
  )

Inc_CS_who_meiru_U <- sum(DEL_meiru$Inc_CS_who_U, na.rm = TRUE) / sum(filter(DEL_meiru, f_id %in% c(101:201, 203))$denom_CS, na.rm = TRUE) * 100000



#################################################################################

CS_table_who <- data.frame(Site = c("Total", "North", "Central", "South", "Urban", "Rural"),
                           CS = round (c(Inc_CS_who_mlw, 0, Inc_CS_who_meiru_C, Inc_CS_who_mlw_S, Inc_CS_who_mlw_U+Inc_CS_who_meiru_U, 0),2))

CS_table_who %>%
  knitr::kable(caption = "Incidence of CS by WHO case definition",col.names=c("Site", "Incidence of CS per 100000 Births")) %>%
  kableExtra::kable_styling(full_width = FALSE)


## ----MAL-CS-----------------------------------------------------------------------------------------------------------------

#for each individual centre
#MLW data
DEL_mlw <- DEL_mlw %>%
  mutate(
    Inc_CS_mal=ifelse((del_syphilis==1 & del_result==1 & del_treatment %in% c(0,9)) |
                      (del_syphilis==1 & del_result==1 & del_treatment==1 & (del_injections %in% c(1:2,9) | (del_injections==3 & del_inj3doc==0))) |
                      ( del_syphilis==1 & del_result==1 & del_treatment==1 & del_injections ==3 & del_inj3doc==1 & del_inj3dt<30), 1, 0)
  )



```

```r
#MEIRU
DEL_meiru <- DEL_meiru %>%
  mutate(
    Inc_CS_mal=ifelse((del_syph==1 & del_result==1 & del_rx==0)  |
                        (del_syph==1 & del_result==1 & del_rx==1 & (del_treat %in% 1:2 |
                        (del_treat==3 & del_dt3doc==0))) |
                        (del_syph==1 & del_result==1 & del_rx==1 & del_treat ==3 &
                        del_dt3doc==1 & del_dt3<30), 1, 0)
  )


Inc_CS_mal_mlw <- (sum(DEL_mlw$Inc_CS_mal, na.rm = TRUE) + sum(DEL_meiru$Inc_CS_mal,
na.rm = TRUE))/ (sum(DEL_mlw$denom_CS, na.rm = TRUE) + sum(DEL_meiru$denom_CS, na.rm =
TRUE)) * 100000


########################################################################################
################

#By region
#North, Central, South

DEL_mlw <- DEL_mlw %>%
  mutate(
    Inc_CS_mal_C=ifelse(del_fid==201 & ((del_syphilis==1 & del_result==1 & del_treatment
    %in% c(0,9)) |
                                          (del_syphilis==1 & del_result==1 &
                                          del_treatment==1 & (del_injections %in% c(1:2,9
                                          ) | (del_injections==3 & del_inj3doc==0))) |
                                          ( del_syphilis==1 & del_result==1 &
                                          del_treatment==1 & del_injections ==3 &
                                          del_inj3doc==1 & del_inj3dt<30)), 1, 0),
    Inc_CS_mal_S=ifelse(del_fid %in% 101:104 & ((del_syphilis==1 & del_result==1 &
    del_treatment %in% c(0,9)) |
                                                  (del_syphilis==1 & del_result==1 & (
                                                  del_injections %in% c(1:2,9) | (
                                                  del_injections==3 & del_inj3doc==0))) |
                                                  ( del_syphilis==1 & del_result==1 &
                                                  del_treatment==1 & del_injections ==3 &
                                                   del_inj3doc==1 & del_inj3dt<30)), 1, 0
                                                  )
  )

#no data from the north and central in the mlw data


Inc_CS_mal_mlw_S <- sum(DEL_mlw$Inc_CS_mal_S, na.rm = TRUE) / sum(filter(DEL_mlw, del_fid
 %in% 101:104)$denom_CS, na.rm = TRUE) * 100000


DEL_meiru <- DEL_meiru %>%
  mutate(
    Inc_CS_mal_N=ifelse(f_id %in% 202:203 & ((del_syph==1 & del_result==1 & del_rx==0)  |
                                              (del_syph==1 & del_result==1 & del_rx==1 &
                                               (del_treat %in% 1:2 | (del_treat==3 &
                                              del_dt3doc==0))) |
                                              (del_syph==1 & del_result==1 & del_rx==1 &
                                               del_treat ==3 & del_dt3doc==1 & del_dt3<
                                              30)), 1, 0),
    Inc_CS_mal_C=ifelse(f_id==201 & ((del_syph==1 & del_result==1 & del_rx==0)  |
                                      (del_syph==1 & del_result==1 & del_rx==1 & (
                                      del_treat %in% 1:2 | (del_treat==3 & del_dt3doc==0
                                      ))) |
                                      (del_syph==1 & del_result==1 & del_rx==1 &
                                      del_treat ==3 & del_dt3doc==1 & del_dt3<30)), 1, 0
                                      )
```

```
  )

#no data from the south in the meiru data

Inc_CS_mal_meiru_N <- sum(DEL_meiru$Inc_CS_mal_N, na.rm = TRUE) / sum(filter(DEL_meiru, f_id %in% 202:203)$denom_CS, na.rm = TRUE) * 100000
Inc_CS_mal_meiru_C <- sum(DEL_meiru$Inc_CS_mal_C, na.rm = TRUE) / sum(filter(DEL_meiru, f_id == 201)$denom_CS, na.rm = TRUE) * 100000

##########################################################################
#By urban vs rural
DEL_mlw <- DEL_mlw %>%
  mutate(
    Inc_CS_mal_U=ifelse(del_fid %in% c(101:104, 201) & ((del_syphilis==1 & del_result==1 & del_treatment %in% c(0,9)) |
                                                                 (del_syphilis==1 & del_result==1 &del_treatment==1 &  ( del_injections %in% c(1:2,9) | (del_injections==3 & del_inj3doc==0))) |
                                                                 ( del_syphilis==1 & del_result ==1 & del_treatment==1 & del_injections ==3 & del_inj3doc==1 & del_inj3dt<30 )), 1, 0)
  )

Inc_CS_mal_mlw_U <- sum(DEL_mlw$Inc_CS_mal_U, na.rm = TRUE) / sum(filter(DEL_mlw, del_fid %in% c(101:201, 203))$denom_CS, na.rm = TRUE) * 100000




DEL_meiru <- DEL_meiru %>%
  mutate(
    Inc_CS_mal_U=ifelse(f_id %in% c(201, 203) & ((del_syph==1 & del_result==1 & del_rx==0 )  |
                                                          (del_syph==1 & del_result==1 & del_rx ==1 & (del_treat %in% 1:2 | (del_treat ==3 & del_dt3doc==0))) |
                                                          (del_syph==1 & del_result==1 & del_rx ==1 & del_treat ==3 & del_dt3doc==1 & del_dt3<30)), 1, 0),
    Inc_CS_mal_R=ifelse(f_id==202 & ((del_syph==1 & del_result==1 & del_rx==0)  |
                                      (del_syph==1 & del_result==1 & del_rx==1 & ( del_treat %in% 1:2 | (del_treat==3 & del_dt3doc==0 ))) |
                                      (del_syph==1 & del_result==1 & del_rx==1 & del_treat ==3 & del_dt3doc==1 & del_dt3<30)), 1, 0 )
  )

Inc_CS_mal_meiru_U <- sum(DEL_meiru$Inc_CS_mal_U, na.rm = TRUE) / sum(filter(DEL_meiru, f_id %in% c(101:201, 203))$denom_CS, na.rm = TRUE) * 100000
Inc_CS_mal_meiru_R <- sum(DEL_meiru$Inc_CS_mal_R, na.rm = TRUE) / sum(filter(DEL_meiru, f_id == 202)$denom_CS, na.rm = TRUE) * 100000

##########################################################################################3

CS_table_mal <- data.frame(Site = c("Total", "North", "Central", "South", "Urban", "Rural"),
                           CS = round (c(Inc_CS_mal_mlw, Inc_CS_mal_meiru_N, Inc_CS_mal_meiru_C, Inc_CS_mal_mlw_S, Inc_CS_mal_mlw_U+ Inc_CS_mal_meiru_U, Inc_CS_mal_meiru_R),2))

```

```
CS_table_mal %>%
  knitr::kable(caption = "Incidence of CS by Malawi case definition",col.names=c("Site",
  "Incidence of CS per 100000 Births")) %>%
  kableExtra::kable_styling(full_width = FALSE)
```
